# Decomposition of Gene Expression State Space Trajectories

**DOI:** 10.1371/journal.pcbi.1000626

**Published:** 2009-12-24

**Authors:** Jessica C. Mar, John Quackenbush

**Affiliations:** 1 Department of Biostatistics, Harvard School of Public Health, Boston, Massachusetts, United States of America; 2 Department of Biostatistics and Computational Biology, Dana-Farber Cancer Institute, Boston, Massachusetts, United States of America; 3 Department of Cancer Biology, Dana-Farber Cancer Institute, Boston, Massachusetts, United States of America; University of Virginia, United States of America

## Abstract

Representing and analyzing complex networks remains a roadblock to creating dynamic network models of biological processes and pathways. The study of cell fate transitions can reveal much about the transcriptional regulatory programs that underlie these phenotypic changes and give rise to the coordinated patterns in expression changes that we observe. The application of gene expression state space trajectories to capture cell fate transitions at the genome-wide level is one approach currently used in the literature. In this paper, we analyze the gene expression dataset of Huang et al. (2005) which follows the differentiation of promyelocytes into neutrophil-like cells in the presence of inducers dimethyl sulfoxide and *all-trans* retinoic acid. Huang et al. (2005) build on the work of Kauffman (2004) who raised the attractor hypothesis, stating that cells exist in an expression landscape and their expression trajectories converge towards attractive sites in this landscape. We propose an alternative interpretation that explains this convergent behavior by recognizing that there are two types of processes participating in these cell fate transitions—core processes that include the specific differentiation pathways of promyelocytes to neutrophils, and transient processes that capture those pathways and responses specific to the inducer. Using functional enrichment analyses, specific biological examples and an analysis of the trajectories and their core and transient components we provide a validation of our hypothesis using the Huang et al. (2005) dataset.

## Author Summary

Understanding how cells differentiate from one state to another is a fundamental problem in biology with implications for better understanding evolution, the development of complex organisms from a single fertilized egg, and the etiology of human disease. One way to view these processes is to examine cells as “complex adaptive systems” where the state of all genes in a cell (more than 20,000 genes) determines that cell's “state” at a given point in time. In this view, differentiating cells move along a path in “state space” from one stable “attractor” to another. In a 2005 paper, Sui Huang and colleagues presented an experimental model in which they claimed to have evidence for such attractors and for the transitions between them. The problem with this approach is that although it is intuitively appealing, it lacks predictive power. Reanalyzing Huang's data, we demonstrate that there is an alternative interpretation that still allows for a state space description but which has greater ability to make testable predictions. Specifically, we show that these abstract state space trajectories can be mapped onto more well-known pathways and represented as a “core” differentiation pathway and “transient” processes that capture the effects of the treatments that initiate differentiation.

## Introduction

Our understanding of the molecular basis of a wide range of biological processes, including development, differentiation, and disease, has evolved significantly in recent years. Increasingly, we are coming to recognize that it is not single genes, but rather complex networks of genes, gene products, and other cellular elements that drive cellular metabolism and cell fate, and when perturbed, can lead to development of disease phenotypes. Representing and analyzing such complex networks, encompassing thousands or tens of thousands of elements, presents significant challenges. One approach that has begun to be applied is the representation of transcriptional changes as transitions that occur with the “state space” defined by the expression states of all genes within the cell [1],[2]. This approach has a number of advantages, including providing a framework for predictive modeling and the incorporation of stochastic components in the biological process.

The underlying assumption in such an analysis is that each cellular phenotype can invariably be traced back to a particular class of genome-wide gene expression signatures representing a specific region of the gene expression state space. As described in Huang et al. [3], this signature for a particular cellular state at a particular instant in time is represented by a multidimensional gene expression vector in a high dimensional space where each coordinate represents the expression level of a particular gene. By considering all possible configurations that this signature can take, we create a multidimensional landscape that is referred to as the expression state space [1]. Each observed phenotype can be represented as a single point in the state space. When cells transition through successive phenotypes, for example, during the different stages of hematopoietic differentiation, specific sets of genes alter their expression levels as dictated by an underlying transcriptional program and these changes can be represented by a continuous trajectory in expression state space; ultimately these represent the transcriptional program being played out by the cell's collection of gene networks and complex pathways.

Kauffman [1] first proposed the idea that stable cell fates, the cellular phenotypes we observe, correspond to “attractors” in the expression state space, stable points to which the system would return to if subjected to a small perturbation. He points out that in principle cells could adopt any permutation of gene expression states (as many as the number of genes and as infinite as the number of expression level states) however this is not what we observe in nature. According to Kauffman, since there are about 250 different cell types, there must be approximately that number of attractors in state space, either valleys or peaks in the landscape, that represent the stable cell fates or cell types that cells will ultimately converge to in the presence of an inducer or perturbation. While this is an interesting model, direct experimental evidence supporting it and its overall utility in explaining cellular mechanism remain to be seen.

Huang et al. [3] reported evidence they claim demonstrated the existence of an attractor. They conducted a gene expression time-course experiment on the differentiation of human HL-60 promyelocytic cells into neutrophils using two different inducers, dimethyl sulfoxide (DMSO) and all-*trans* retinoic acid (ATRA). Time-course data was collected using Affymetrix U95Av2 GeneChips and analyzed to provide gene expression level measures necessary to create a state-space model. Using principal components analysis, they develop a two-dimensional state space representation in which DMSO and ATRA induce initially divergent trajectories that, over time, converge on a common trajectory leading to a final expression state representing the neutrophils. They argue that instead of observing trajectories that explore the state space, the trajectories display convergence to a single point and that this therefore provides empirical proof that attractive states exist in nature.

Here, we propose an alternative interpretation of this convergent behavior that does not appeal to the attractor hypothesis but rather explores this observation in the context of a superposition of components that reflect the pathways activated by the applied perturbations. To this end, we extend the work of Huang et al. [3] by decomposing the state space trajectories into components comprising two sets of genes, a core group and transient group that capture the stimulus-independent and stimulus-dependent effects, respectively. The superposition of these components reflect the observation that both sources of effects independently influence the overall shape of the trajectory taken during the cell fate transition. We show how this division allows us to look at functional behavior of genes and their contribution to the cell fate transitions in a more enlightening way. Using regression models, we isolate core genes that are common to both stimuli and represent those critical to the differentiation process. The genes outside the core represent the transient component of the trajectory corresponding to the perturbation effects. To illustrate our ideas, we apply our method to the same published dataset generated by Huang et al. [3].

The HL-60 cell line has long been used as a model to understand the molecular mechanisms driving the progression and pathogenesis of acute promyelocytic leukemia (APL) [4]. In normal promyelocytes, proliferation and differentiation are tightly coupled processes. However this balance comes unstuck in APL cells and as a result cells proliferate in a disregulated fashion. The discovery that inducers like RA and DMSO could reprogram APL cells to overcome this block and resume differentiation, led to the emergence of a class of therapeutics known as differentiation therapy [5].

DMSO is an organic solvent but also functions as a cryoprotective agent for tissue cell culture [4]. Although it is widely used in veterinary medicine in the treatment of pain and inflammation, it is not generally used in humans because it is known to be hepatotoxic. The hormone ATRA is a derivative of vitamin A and belongs to a class of molecules called retinoids [6]. ATRA is currently used in differentiation therapies that treat human patients with APL. Current complete remission rates for APL patients on ATRA-based differentiation therapy in combination with chemotherapy have been reported to be as high as 90–95% [7]. At the molecular level, both DMSO and ATRA arrest the cell cycle at the G1-S phase transition point, and induce terminal differentiation of HL-60 cells, resulting in neutrophil-like cells.

ATRA and DMSO are biochemically distinct molecules that activate slightly different sets of pathways in HL-60 cells. Huang et al. [3] explain that this is the reason why the trajectories initially diverge and explore different parts of the expression state space. They argue that it is the presence of an attractor that then causes the trajectories to converge from different directions to eventually arrive at a common endpoint, and discount the possibility of a “specific, unique differentiation pathway” that may be triggered by both inducers.

While this argument may seem conceptually appealing, upon further inspection the attractor hypothesis greatly limits our ability to develop mechanistic interpretations or to build predictive models of cell fate transitions. We believe that there exists an alternative, more plausible interpretation that Huang et al. [3] and Kauffman [1] have not considered. Our interpretation is based on the recognition that there are two types of processes that contribute to cell fate transitions: one, a core biological process inherent to the transition-specific event and two, a transient process related to the direct effects that the particular inducing agent exerts on the cell. The early divergence seen in the state space trajectories described by Huang et al. [3] is reflective of the cells' response to specific perturbation and the compound-specific response that follows. We expect these transient processes to dominate only at the initial period of the time-course since most drugs are metabolized quickly by the cell. Once this disorder has subsided, the targeted effects of each inducer are expected to have begun triggering the core processes and as this occurs, the directions that both trajectories adopt become more and more convergent because the overlap in activated pathways in DMSO-induced cells and ATRA-induced cells is growing larger as the cells transition towards their common endpoint. The source of this convergence therefore is not necessarily due to the existence of an attractor but instead can be explained by the combination of these two types of processes exerting their temporal effects on cells. Indeed, if such an attractor existed, then there should be a whole class of perturbations that would cause transitions from the initial to the final state, rather than a small number that activate a single core pathway. If one adopts the attractor hypothesis as the basis for cell-fate transitions, then our interpretation is much closer to that of Conrad Waddington, in which he argued for the “canalization” of state space through the existence of defined paths, or canals, between attractor states [8]–[10].

## Results

### Preprocessing of the Gene Expression Time-Course Dataset

The Affymetrix U95Av2 GeneChips used by Huang et al. [3] gave an original dataset with approximately 12, 600 genes, measured at twelve time points: two hours, four hours, eight hours, twelve hours, eighteen hours, one day, two days, three days, four days, five days, six days, seven days post-stimulation with ATRA and DMSO. Filters were applied to this dataset to remove genes that were associated with low expression or did not show significant expression changes across the time points measured. 3841 genes were retained by this filtering process. The expression measures in the dataset provided by Huang et al. [3] are represented as normalized log_2_ expression ratio values where each gene's ratio is formed by comparing its expression measure in the stimulated time-course to its corresponding expression in a non-stimulated control sample of HL-60 cells.

In addition to the preprocessing steps already taken by Huang et al. [3], it was necessary to remove genes that were considered flat, that is genes that showed no change in their expression profiles across the entire duration of the experiment for both inducers. Since these genes clearly do not play a regulatory role in the differential transcriptional program, it was necessary to remove them from our analyses because their inclusion would only dilute out meaningful results. Given that there were twelve time points available, we fitted a cubic polynomial regression model separately for each gene's expression profile under each stimulus. For example, for a single gene: 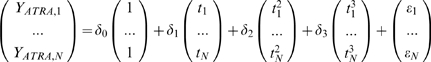


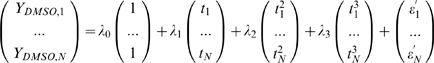
where *Y_ATRA,t_* and *Y_DMSO,t_* are the expression measures at time *t* under ATRA and DMSO respectively; 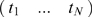 corresponds to the measured time points (N = 12 for the Huang et al. dataset) and 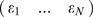 and 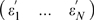 represent random Normal residual error terms.

A gene was discarded if both models (under DMSO and ATRA) were not significant at the 0.05 level. Using this approach, 951 genes were filtered out from a starting total of 3841 genes.

It is worth nothing that the model fit for some expression profiles might be improved by using polynomial regression models of higher order than the cubic model we have adopted. However this also represents an additional layer of parameter estimation, and for reasons of parsimony we have applied only the cubic model for all genes in our dataset.

### Dividing Genes into Mutually Exclusive Groups Based on their Role in the Cell Fate Transitions

**Figure 1:**
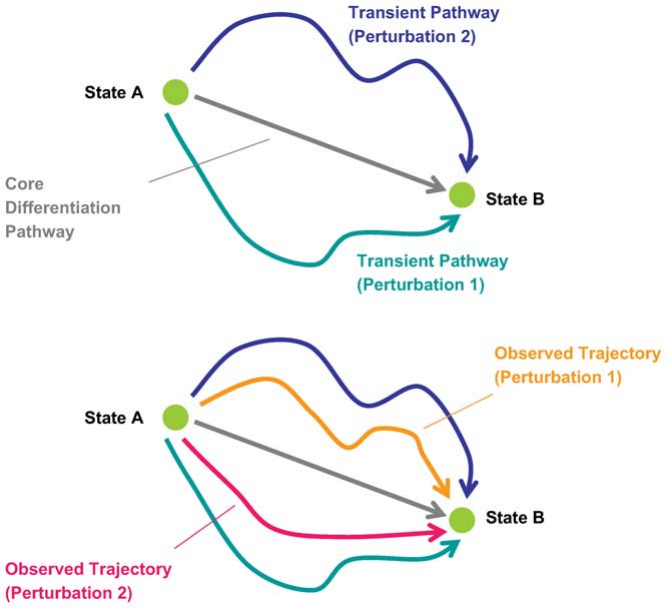
Schematic diagram outlining our hypothesis.

Our analysis is based on the simple hypothesis regarding the state space trajectories governing the observed cell fate transition, namely that any observed trajectory can be decomposed into two independent parts (see Figure 1); one component represents the changes inherent to the specific biological process driving the cell fate transitions (e.g. differentiation of promyelocytes) and the other component captures the transient effects of the cell's direct response to the perturbation (e.g. metabolism of DMSO or ATRA). We use the terms core group and transient group to distinguish these two sets respectively.

Based on this hypothesis, one should be able to define the set of core genes based on comparison of expression profiles under different stimuli. The core genes are those response genes that are in common between stimuli and which are highly correlated. The assumption is that such genes represent the specific differentiation pathways carrying the cells between their initial and final states. Expression profiles of a core gene are therefore expected to be fairly robust for different perturbations. Similarly, the transient genes are those which differ between the two stimuli and which likely represent the metabolic processing of the stimulating agent, as well as any short-term changes induced by stimuli unrelated to changing the cell fate. Identifying these genes simply requires identifying those genes with altered patterns of expression but which are not well correlated between stimuli.

The formation of core and transient groups is based on a data-driven classifier that is applied to the time-course gene expression data. Our classification scheme begins first by fitting cubic regression models to each individual gene expression profile. A cubic model was chosen for this dataset because of the moderately large number of time points available to fit a model with four to eight covariates. For a single gene, both a full model and a reduced model are fitted to its time-course expression profiles for each perturbation. The full model specifies a set of parameters that capture the time-dependent curvature of a gene's expression profile for each separate perturbation. In this way, the full model assumes that the expression profile is different across the two perturbations.

For a single gene, the full model is specified by the following formulae, where *Y_a,b_* denotes the gene expression value measured for inducer *a* at time point *b*:
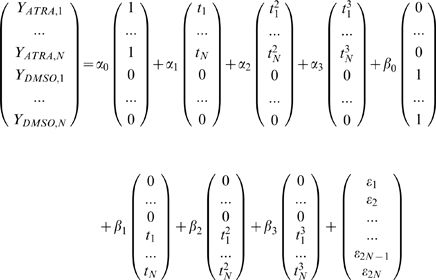


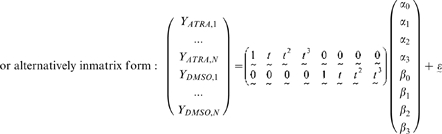
where 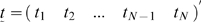 corresponds to the measured time points (N = 12 for the Huang et al. dataset) and 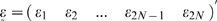 represents a random Normal residual error term.

The parameters *α_0_*, *α_1_*, *α_2_*, *α_3_* and *β_0_*, *β_1_*, *β_2_*, *β_3_* are the coefficients of the time covariate in the model for the ATRA-induced and DMSO-induced time series respectively. We interpret these parameters in the following way: *α_0_* represents the gene's expression in the ATRA-induced time series at time zero, *α_1_* represents the linear time effect on the gene's expression in the ATRA-induced time series, similarly *α_2_* and *α_3_* represent the quadratic and cubic time effects, respectively. Essentially these parameters measure the effect that the time component has on a gene's expression level, and we allow for the possibility of time having a polynomial effect up to degree three.

The reduced model is a simpler model that assumes the expression profiles for different perturbations need only be specified by the one set of parameters.

For a single gene, the reduced model is specified by the following formulae: 
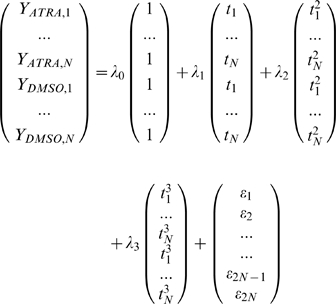


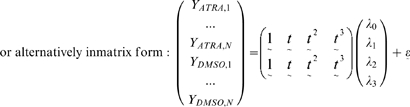
where 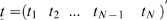 and 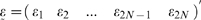 as defined in the full model.

Fitting these two models to a single gene, is equivalent to proposing two hypotheses: one, that this gene belongs in the core group and is therefore defined by the reduced model, and two, this gene belongs in the transient group and is defined by the full model. To decide which of these two hypotheses is more plausible given the available data, we use the analysis of deviance test, which is an extension of the likelihood ratio test.

The likelihood ratio statistic calculates the likelihood function (in other words how well the data fits a hypothesized model) under two different hypotheses and evaluates how statistically significant the difference between the two likelihood functions is.

The likelihood ratio statistic Λ(*x*) takes the form of:


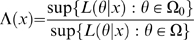
 where 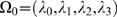 represents the parameters specified under the null hypothesis that the gene belongs in the core group and its profile is explained by our reduced model.

The second vector 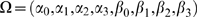 represents the parameters specified under the alternative hypothesis that the gene belongs in the transient group and is therefore is specified by the full model.

*L*(*θ* | *x*) is the likelihood function specified by the model. The numerator of Λ(*x*) represents this likelihood function calculated under the reduced model (the core gene hypothesis), we use the data to find the estimates of the unknown parameters that maximize this likelihood. 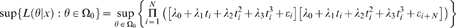


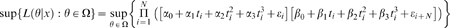



The statistic -2log_2_[Λ(*x*)] is asymptotically distributed as a Chi-square random variable with four degrees of freedom. Therefore at the 0.05 level of significance, if this statistic is greater than 3.841, this implies that the difference between the two likelihoods is significant and the full model has a higher likelihood, given the expression profile data available. A gene with this result would hence be placed in the transient group.

An extension of this test is the analysis of deviance, which instead compares the size of the likelihood ratio statistic with the likelihood of the full model alone. Because the full model specifies more parameters, it follows that the likelihood of the full model will be higher than the likelihood under the reduced model. What we are interested in testing then is whether the improvement in the model fit obtained with the full model over the reduced model is statistically significant.

The analysis of deviance uses the ratio of the likelihood ratio statistic and the likelihood under the full model only. This ratio statistic is distributed as an *F* random variable with 4 and 16 degrees of freedom. The rationale is that when the full model results in a very large improvement over the reduced model, this ratio statistic will be very large and hence the P-value will be very small. This suggests that the full model significantly improves the model fit to the expression profile data and the associated gene will be classified as a transient gene. For more details on how the likelihood ratio statistic and the analysis of deviance are computed from the data, please see Text S1 [11].

Because our method involves testing almost three thousand hypothesis tests, the chance of detecting a false positive result grows to a non-trivial degree, for example at the 0.05 level, we expect to declare about 144 transient genes purely by chance. To correct against this, the resulting P-values must be adjusted for multiple testing. We have chosen to adjust our P-values using the Benjamini-Hochberg method which controls the false discovery rate [12]. Genes with significant adjusted P-values (less than 0.1) are placed in the transient group; all other genes are in the core group. For the Huang et al. dataset, this classification scheme identified 1428 core genes and 1462 transient genes (see Tables S1 and S2) [11].

One limitation of this modeling approach is that for some genes, there is a low degree of similarity between the observed expression profile and the one predicted by the most appropriate model (see Figure S1) [11]. This is especially the case for genes that display spiky expression profiles across the time series. This is partly due to the low degree of temporal sampling of what is a complex dynamic system but also in part due to potential biological differences in the samples themselves. It is possible that other curve-fitting methods such as a Fourier transform or spline-dependent algorithm might also be applicable to this kind of data. However these methods do not provide the statistical machinery that comes with the regression modeling approach that we have taken. The main advantage of our method is being able to easily apply valid statistical tests to determine which one of two models is more likely in light of the data. We are also able to explicitly define statistical significance in a meaningful way that protects against a specified false discovery rate.

### Functional Enrichment Analysis

Our interpretation of the convergent behavior seen in the expression states of cell fate transitions hinges on the assumption that genes can be divided into a core or transient group, based on their functional role in the cell fate transition. For this assumption to be valid, we would therefore expect the genes in the core group to be involved in processes related to the differentiation of promyelocytes. Similarly, the transient group is expected to have genes involved in processes related to the metabolism of the inducer and a general response to the exposure of a foreign stimulus. To investigate whether there is any evidence in the data to support our assumptions, the core and transient groups were subjected to a representation analysis using their GO term assignments. The Gene Ontology Project [13] attempts to classify gene products, assigning proteins to groups specifying their Molecular Function, the Biological Process to which they contribute, and their Cellular Component [14]. The GO terms in each class form a hierarchy of increasing specificity (formally a directed acyclic graph or DAG) so that the broadest classifications provide a general picture of the functional class to which a gene belongs (for example, a kinase) while more precise terms will specify precisely what a particular gene does (such as specifying the substrate on which a kinase acts). Functional category over-representation was assessed using the Fisher's exact test with a Benjamini-Hochberg correction to adjust for multiple testing; P-values were retained at the 0.1 significance level.

We identified 13 GO functional classes that were over-represented in the core group, relative to the transient group. All of these classes were associated with transcription and RNA metabolism (see Table 1). These results support our assumption that the genes in the core group are associated with a common differentiation process. This can be seen by considering the over-enrichment of GO categories for transcription, and transcriptional regulation. During differentiation, cells require increased access to a diverse range of proteins to transform themselves into new cell types. The synthesis of these proteins can only come about through the differential expression of key transcriptional networks which based on the results of our functional enrichment analysis, clearly affect a significant proportion of those genes found in the core group. The fact that we did not see categories more specific to the differentiation of promyelocytes into neutrophils may be because these highly-specific terms usually sit at the periphery of the GO hierarchy and only a small number of genes are assigned to these functional classes. Therefore, it is less likely to see enrichment given the starting pool of genes retained by the filtering procedures. For example, neutrophil differentiation class has five gene products, regulation of neutrophil differentiation class has two gene products in the current version of GO.

**Table 1:** Enriched GO terms for the core group that were statistically significant at the 0.1 level.

GO ID	Number of Core Genes in GO Term	Total Number of Core Genes	Total Number of Transient Genes	Number of Genes in GO Term	P-value	Adjusted P-value	Ontology	GO Term
GO:0003676	358	1428	1462	618	1.08×10^−6^	0.00833	MF	Nucleic acid binding
GO:0016070	317	1428	1462	547	5.52×10^−6^	0.0129	BP	RNA metabolic process
GO:0044446	339	1428	1462	590	7.10×10^−6^	0.0129	CC	intracellular organelle part
GO:0044422	340	1428	1462	592	7.24×10^−6^	0.0129	CC	organelle part
GO:0044428	168	1428	1462	271	8.41×10^−6^	0.0129	CC	Nuclear part
GO:0006350	257	1428	1462	441	3.18×10^−5^	0.0407	BP	transcription
GO:0032774	234	1428	1462	400	5.46×10^−5^	0.0460	BP	RNA biosynthetic process
GO:0032991	271	1428	1462	470	5.60×10^−5^	0.0460	CC	macromolecular complex
GO:0006694	17	1428	1462	18	5.74×10^−5^	0.0460	BP	steroid biosynthetic process
GO:0006351	23	1428	1462	397	6.53×10^−5^	0.0460	BP	transcription, DNA-dependent
GO:0006355	224	1428	1462	382	6.58×10^−5^	0.0460	BP	regulation of transcription, DNA-dependent
GO:0045449	239	1428	1462	413	0.000124	0.0792	BP	regulation of transcription
GO:0006139	430	1428	1462	782	0.000152	0.0902	BP	nucleobase, nucleoside, nucleotide and nucleic acid metabolic process

The enriched GO functional classes in the transient group, relative to the core group, showed compelling evidence in line with our assumption that transient genes are primarily involved in perturbation-related processes only. We identified seven enriched GO functional classes, and all seven collectively describe typical cellular responses to an external perturbation (see Table 2). For instance, “defense response”, “response to external stimulus”, “response to wounding” (defined as “A change in state or activity of a cell or an organism (in terms of movement, secretion, enzyme production, gene expression, etc.) as a result of a stimulus indicating damage to the organism.”) and “response to stimulus”, “inflammatory response” (defined as “The immediate defensive reaction (by vertebrate tissue) to infection or injury caused by chemical or physical agents.”) are exactly the kind of functional classes we would expect to see based on our definition of the transient gene group. The remaining two classes “signal transduction” and “cell communication” describe processes that are directly triggered by an inducer.

**Table 2:** Enriched GO terms for the transient group that were statistically significant at the 0.1 level.

GO ID	Number of Core Genes in GO Term	Total Number of Transient Genes	Total Number of Core Genes	Number of Genes in GO Term	P-value	Adjusted P-value	Ontology	GO Term
GO:0006952	99	1462	1428	137	1.02×10^−7^	0.000471	BP	defense response
GO:0009605	92	1462	1428	126	1.32×10^−7^	0.000471	BP	response to external stimulus
GO:0009611	73	1462	1428	96	1.84×10^−7^	0.000471	BP	response to wounding
GO:0006954	60	1462	1428	77	5.42×10^−7^	0.00104	BP	inflammatory response
GO:0007165	373	1462	1428	638	3.89×10^−6^	0.00542	BP	signal transduction
GO:0050896	291	1462	1428	486	4.23×10^−6^	0.00542	BP	response to stimulus
GO:0007154	393	1462	1428	679	8.24×10^−6^	0.00906	BP	cell communication

### Specific Biological Examples

**Figure 2:**
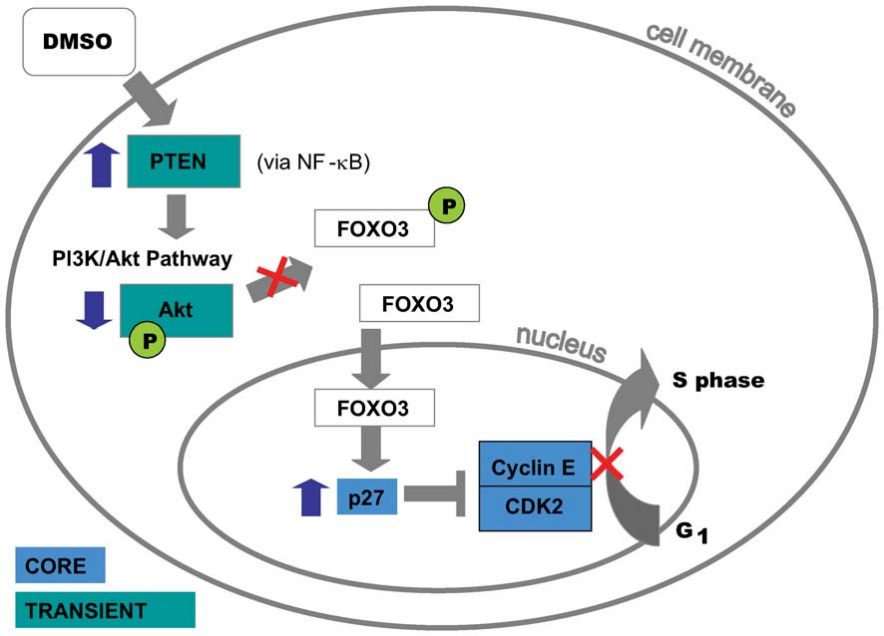
DMSO signaling pathway. DMSO upregulates the tumor suppressor protein PTEN [15] in HL-60 cells. This increase in PTEN expression and activity is brought about by the activation of NF-κB. PTEN is a lipid phosphatase that is located in the cytoplasm and one of its primary roles is to dephosphorylate PIP3, a product of PI3K. The upregulation of PTEN results in a perturbation of the PI3k/Akt pathway, specifically the reduction in Akt phosphorylation levels and hence decreasing the amount of activated Akt. Normally activated Akt leads to phosphorylation of FOXO3, a member of the forkhead transcription factor family and this sets off further pathways that promote cell survival. However, inactive FOXO3 is able to translocate to the nucleus where it acts as a transcription factor, binding to *cis*-DNA elements and causing an increase in the gene expression of p27 [16]. The p27 protein inhibits the cyclin-dependent kinase complex Cyclin E and CDK2 which controls the G1 to S phase transition.

As a further validation step of our proposed model, we utilize the information known about the induction pathways of DMSO and ATRA in HL-60 cells since we expect these genes whose expression is regulated by these pathways to be in the transient group. The proteins PTEN, Akt1, p27 play an integral role in the signaling pathways triggered directly by DMSO [15],[16] (see Figures 2 and S2 [17]). The genes corresponding to these proteins are known to be differentially expressed by DMSO. PTEN and Akt were classified as transient genes by our model.

**Figure 3:**
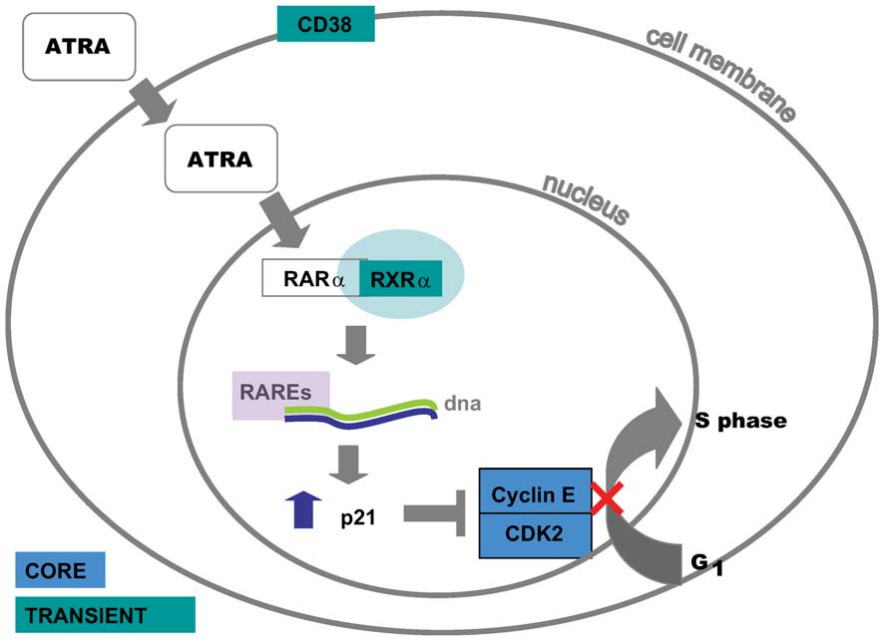
ATRA signaling pathway. ATRA is able to diffuse freely across the cell membrane. A pair of cellular retinoic acid binding proteins act as cell surface receptors for retinoids however these have been shown to be dispensable in retinoic-acid signaling [21]. ATRA binds to a family of nuclear hormone receptors called retinoic-acid receptors (RARs). There are three subtypes of the RAR family, these are encoded by different genes and denoted RAR-α, RAR-β, RAR-γ. Collins et al [22] demonstrated that in HL-60 cells, ATRA induced granulocytic differentiation by binding RAR-α directly. RAR-α binds to specific *cis*-acting DNA sites, known as retinoic-acid response elements (RAREs). These RAREs are located in the promoter sequences of specific genes that are targets of RAR-α. In order to bind DNA efficiently, RARs must however form heterodimers with a second family of nuclear hormone receptors, the retinoid X receptors (RXRs), of which there are three subtypes: RXR-α, RXR-β, RXR-γ. Both RXRs and RARs function as ligand-dependent transcription factors. One of the RAR-target genes whose expression is upregulated is the cell cycle protein p21 [23]. p21 inhibits the cyclin dependent kinase complex Cyclin E and CDK2. In this way, ATRA induces cell cycle arrest at the G1 to S phase transition checkpoint.

Similarly, we saw key ATRA-induced genes: RXR-α, p21, CITED2, RARRES3, MBN, CD38 and SMYD5 featured in the transient group [18]–[23] (see Figures 3 and S3 [17]). CITED2 is the CREB binding protein/p300 complex that is a transcriptional activator that is induced by ATRA [18]. RARRES3 is one of three known genes that respond to the synthetic retinoid tazarotene, a common treatment for dermatological diseases (the other two genes do not feature in our data). The myeloblastin gene (MBN) is known to be down-regulated during RA-induced differentiation of HL-60 cells. MBN is a serine proteinease, also called Proteinase-3. SMYD5 is a member of the SMYD family and its expression also responds to RA induction [19]. HL-60 cells normally do not express the cell surface antigen CD38, but when exposed to ATRA, these cells undergo an immunophenotypic transition to become CD38+ [20].

In surveying the literature, we were able to identify a total of ten genes whose expression levels have been reported to be induced by either DMSO or ATRA. This includes three induced by DMSO (Akt, p27, PTEN) and seven by ATRA (RXR-α, p21, CITED2, RARRES3, MBN, CD38, SMYD5). Of these ten, only one (p27) was not identified as belonging to the transient group using our approach. Therefore, observing nine out of ten inducer-related genes in the transient group was a statistically significant result with a P-value of 0.0117 (Fisher's exact test, see Text S2 [17]).

Our model also predicts that key genes involved in the differentiation of promyelocytes should feature in the core group. We took the set of sixteen genes identified by two papers that studied the myeloid-specific differentiation pathways in HL-60 cells; these genes belonged to the Myc, Mad, Bcl-2 and Caspase families [24],[25] (see Figure S4 [17]). Thirteen out of sixteen were classified as core genes. This result was highly significant with a P-value of 0.00924,

The Myc proteins are a family of transcription factors that regulate important cellular processes like proliferation, apoptosis and differentiation. HL-60 cells are naturally in a proliferative state, however in the presence of an inducer like ATRA or DMSO, the transition to enter the differentiation pathway is brought about by Myc and Mad [24]. Myc, specifically c-myc is abundant in proliferating HL-60 cells, but its downregulation is associated with differentiating HL-60 cells. Mad is a family of mitotic checkpoint genes and their expression prevents a cell from completing the cell cycle. Mad1 mRNA transcripts are highly expressed in differentiating HL-60 cells but undetectable in proliferating cells. Members of the Myc family and the Mad family with expression data available were: c-Myc, Myc binding protein 2 (a nuclear protein that binds specifically to Myc) [26] and Mad2. These three genes were in our core group.

Upon differentiation, HL-60 cells undergo apoptosis [25]. To this end, HL-60 cells are known to downregulate genes in the Bcl-2 family which promotes cell survival, and upregulate genes in the caspase family which mediate cell death. Expression data was available for the following Bcl-2 genes: Bcl-2, Bfl-1 (BCL2A1), Bik, Bcl-w (BCL2L2), Bax, BCLAF1 (Bcl2-associated transcription factor 1); and for the following caspase genes: Caspase-1, Caspase-2, Caspase-3, Caspase-6, Caspase-8, Caspase-9, Caspase-10. All the Bcl-2 genes were in the core group, except for Bik. Five out of the seven caspases were in the core group, while Caspase-1 and Caspase-3 were in the transient group. Having 10 out of the 13 apoptosis-related genes in the core group also represented a statistical significant enrichment (Fisher's exact P-value 0.0418).

Based on our understanding of the mechanisms of DMSO and ATRA signaling in HL-60 cells, we know that the point at which these pathways converge is at the G1-S phase transition point controlled by the cyclin dependent kinase complex of Cyclin E and CDK2. The genes that encode Cyclin E and CDK2 were both placed in the core group.

By pulling out known genes that play a critical role in the DMSO and ATRA signaling pathways we see that most of these genes are in the transient group. Similarly the majority of the sixteen genes in the myeloid differentiation pathway were in the core group. These examples provide further evidence to support our explanation that underlying these cell fate transitions, there is an interplay between a transient pathway and a core pathway that involves those genes that regulate inducer-specific and differentiation-specific processes, respectively. One limiting factor in our analysis was the restriction imposed by the starting pool of 3841 genes retained from filtering steps applied by Huang et al [3]. There were several canonical genes (such as RAR-α, Mad1) that were not in this group of 3841 genes and therefore were not included in the downstream analyses.

### Expression Trajectories

Visualization of the gene expression changes as they occur over the duration of the time-course shows how this overall signal can be decomposed into the transient and differentiation-specific components. We constructed heatmap representations of the trajectories using the visualization software tool GEDI ([27], see Figure S5 [11]). This tool displays dominant patterns in high dimensional gene expression data by applying a self-organizing map (SOM) clustering algorithm and then creating mosaic tiles which are colored according to a map that represents the centroid values of each gene cluster. In this way, mosaics can be constructed for each time point or each sample in the experiment, and the expression pattern changes occurring across the experiment are visually highlighted.

**Figure 4:**
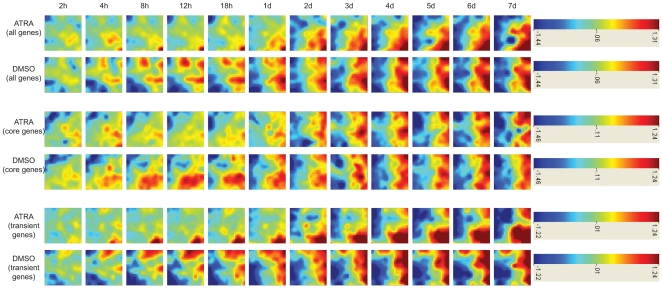
Gene expression mosaics of the ATRA and DMSO-stimulated time course data. The expression mosaics for the ATRA and DMSO-stimulated time course data capture spatial patterns in the data as the system iterates through the time series. These images are a graphical representation of dynamic expression changes in the data, clustered using a self-organizing map algorithm (SOM). We show how the overall expression trajectories for the ATRA and DMSO-stimulated data can be divided into components defined by the core and transient set of genes. Red denotes extreme positive log expression ratios, blue denotes extreme negative log expression ratios.

Figure 4 shows the heatmap representation for the separate trajectories formed for the core and transient groups and the overall group of 2980 genes. The SOM used by the GEDI tool had a grid of 25 rows and 26 columns. On average, each tile contains about 5 genes for the overall group and about 3 genes for the core and transient component groups. We can see how the overall trajectory initially displays divergence between the ATRA and DMSO signal. After 2 days however, the trajectory begins to converge. The transient group trajectory displays heatmaps that for the DMSO and ATRA signal are almost inverted, mirror images of each other. The core group trajectory on the other hand displays heatmaps that have highly similar structures for DMSO and ATRA for the duration of the time-course. We also constructed the core and transient-specific components of the gene expression trajectories using principal component analysis (see Figure S6 [17]).

## Discussion

Using the HL-60 cell line as an example, we note that the main molecular outcome of the two inducers DMSO and ATRA, is to arrest the cell cycle at the G1-S phase transition point. DMSO exerts this effect ultimately by increasing the expression of p27 whereas ATRA upregulates the expression of p21. Both of these proteins p27 and p21 block the cyclin dependent kinase complex Cyclin E and CDK2 which normally drives the cell cycle past this transition point. HL-60 cells are therefore stimulated to converge to a state where both cell populations exhibit the genome-wide gene expression profiles associated with an arrested cell cycle (and thereby the differentiation profiles associated with neutrophil-like cells), however it is important to point out that this convergence is an eventual result of the molecular effects exerted by the inducers. This convergence only emerges later in the time-course because these inducers have different biochemical means of initially getting to the core differentiation pathway. By recognizing this interplay between the two types of processes that are driving cell fate transitions: a transient group and a core group, we can provide a solid link between our knowledge of key molecular events occurring in this system with the convergent properties of these trajectories that we are observing.

Attempts to reconcile the attractor hypothesis with an equally robust explanation that appeals to molecular biology are difficult and reveal some of the limitations of the attractor hypothesis. Specifically, it does not afford the mechanistic interpretation that the core/transient profile decomposition does. While it is worth noting that the temporal damping of the transient and core differentiation gene expression levels is consistent with what one would expect in the case of an attractor, the attractor hypothesis in its simplest form suggests that there should be a large class of perturbations that displace a biological system from its initial state, initiating the transition to its final state. This is in stark contrast to what we observe biologically [28].

The resolution to this apparent contradiction may be that the landscape of the gene expression state space is much more complex and “rugged” than most simple models assume. Conrad Waddington first proposed the notion of “canalization” (essentially, the existence of canal-like routes) in describing cell fate transitions to describe what he saw as a relatively small number of allowable state space trajectories connecting initial and final cell states [8]. Waddington used the analogy of water flowing down a hill via a series of valleys which correspond to these canalized paths which would, by their nature, be robust to small perturbations. In much the same way, the core and transient components suggested by our trajectory model can be thought of as being a “core” component equivalent to the central pathway between states with the transient components representing orthogonal perturbations relative to the core downhill pathways. In such a model, one could imagine selective pressure over time defining the canals as particular pathways become increasingly essential to allow transitions to well-defined cellular states. In such a model, one could argue that the development of undifferentiated states, such as those which develop in cancer, arise when the canals are destroyed or significantly altered. In any event, the overall effect of this combination of core and transient components is that differentiation pathways are buffered against perturbations but are still able to mediate apparently deterministic transitions between phenotypes.

It may be argued that by our definition of what makes a core and a transient process, we are entering a circular argument and imposing structural properties on the trajectory components that we originally hypothesized we might see. For example, because core genes are defined as those whose expression profiles remain invariant for different inducers, we would consequently expect that the core trajectories for two inducers to have similar shapes, and the transient trajectories would be less comparable. We acknowledge that this is somewhat true, however we believe that the true power of our approach lies in the framework that it provides in allowing us to deconvolute high-throughput data on perturbed networks. By being able to resolve the transient, perturbation-driven processes from the core pathways, this approach gives us a means to compare the effects of different perturbations on a systems-level.

For example, in a situation where a chemotherapeutic drug results in differential remission rates amongst patients, we may begin to explore how this same perturbation applied to multiple patients affects these transient and core components. The current framework also provides a means to extend this model to explicitly acknowledge the role stochastic processes play in the cell. We could model the trajectory components as realizations of a stochastic process and construct an appropriate probability distribution or density function that describes this process. Such a model allows for deviations from the most likely route and consequently allows for changes that could lead to the transition from normal cells to the development of disease states. Such models would have applications in understanding the development of disease states such as APL or in understanding systems-level evolution of phenotypic responses such as drug resistance. Using our probability distribution model, we could make predictions of the most likely trajectory a cellular system will take in consideration of external cues or microenvironment properties of the system.

## Methods

### Annotation Sources

We made use of the mappings to GO categories and KEGG pathways from the latest version at the time of the Bioconductor annotation package hgu95av2 (version 2.0.1).

### Data Availability

The full set of expression data is made publicly available through GEO (accession identifier: GSE14500). The expression data for the 3841 genes can be downloaded as a supplemental file (Dataset S1) and is also made available from our website [11].

## Supporting Information

Dataset S1 This file contains the gene expression data for the 3841 genes that were retained after filters were applied to remove genes that were associated with low expression or did not show significant expression changes across the time points measured. The expression measures in the data set provided by Huang et al. [3] are represented as normalized log2 expression ratio values where each gene's ratio is formed by comparing its expression measure in the stimulated time-course to its corresponding expression in a non-stimulated control sample of HL-60 cells.
(1.35 MB XLS)

Figure S1 CD4 is an example of a gene with a spiky expression profile and our model does a limited job at predicting the expression levels observed. However, the purpose of our model is not to predict expression but to estimate parameters that lets us determine whether a particular gene belongs in the core or transient group within a robust statistical framework that gives us the means to adjust for false positives and multiple testing issues.
(0.02 MB PDF)

Figure S2 Expression profiles for some genes involved in DMSO-induced signaling.
(0.03 MB PDF)

Figure S3 Expression profiles for some genes involved in ATRA-induced signaling.
(0.03 MB PDF)

Figure S4 Expression profiles for some genes known to participate in myeloid differentiation.
(0.03 MB PDF)

Figure S5 Gene expression trajectories and their core and transient sub-components for the DMSO and ATRA-stimulated data.
(0.02 MB PDF)

Figure S6 Cartoon describing how Figure 3 was constructed using GEDI software.
(0.02 MB PDF)

Table S1 The core genes. Table S1 lists the 1428 genes that were placed in the core group.
(0.06 MB PDF)

Table S2 The transient genes. Table S2 lists the 1462 genes that were placed in the transient group.
(0.06 MB PDF)

Text S1 The Likelihood Ration Test. Text S1 contains information on how the likelihood ratio test and the analysis of deviance test are computed from the data. This section includes an example using expression data for a gene to show how our method uses these tests to places genes in the core and transient groups.
(0.04 MB PDF)

Text S2 Using Fisher's exact test. Text S2 outlines how we use the Fisher's exact test to compute the significance of seeing an enrichment of transient or core genes in sets of genes extracted from the literature.
(0.02 MB PDF)
