# Supplementary figures and images for: Decomposition of Gene Expression State Space Trajectories
Source: PLoS Comput Biol. 2009 Dec 24;5(12):e1000626. doi: 10.1371/journal.pcbi.1000626 (PMC2791157; doi:10.1371/journal.pcbi.1000626)

**Gene Expression Profile of CD4**

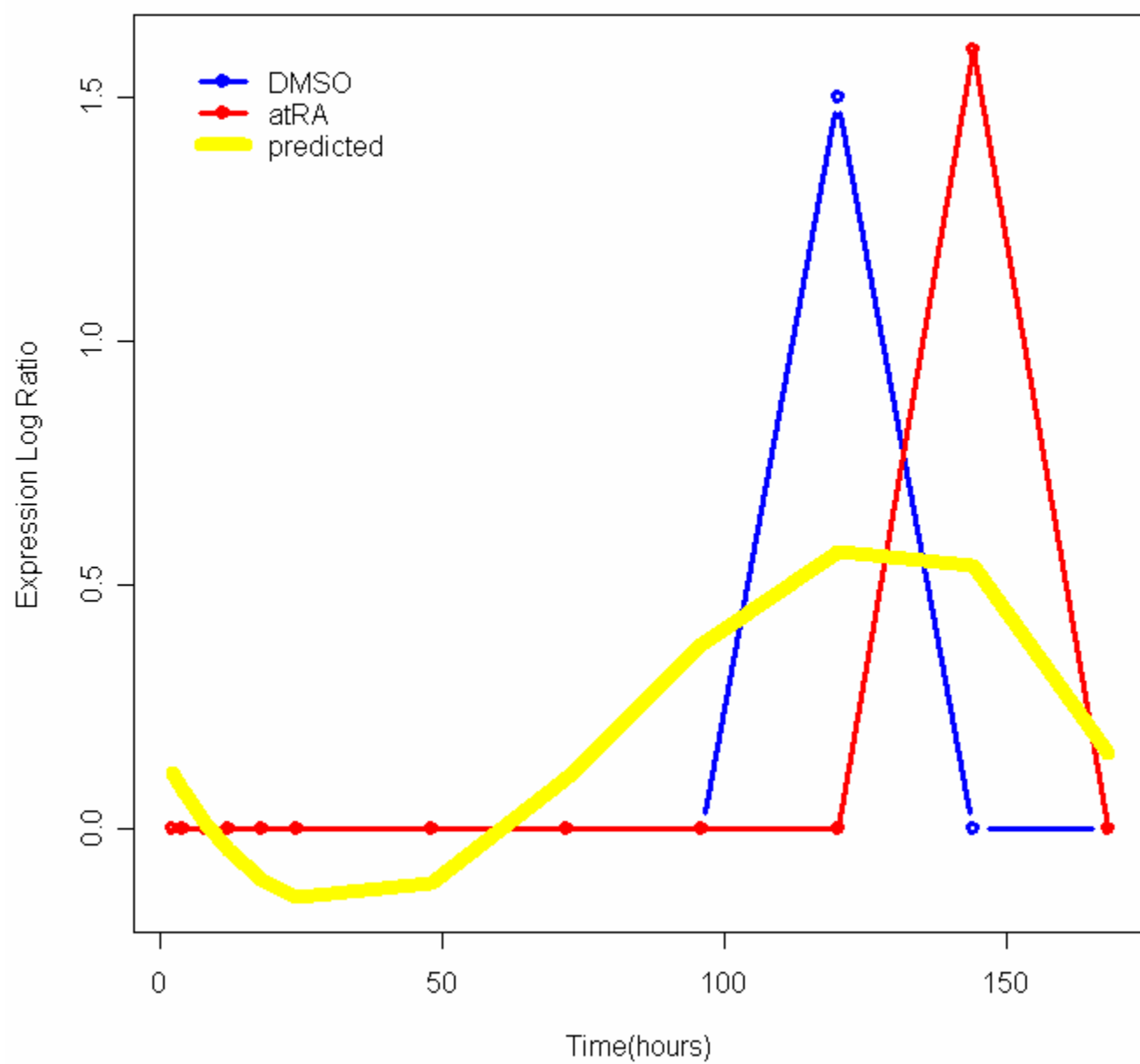

Supplement: Figure S1 — CD4 is an example of a gene with a spiky expression profile and our model does a limited job at predicting the expression levels observed. However, the purpose of our model is not to predict expression but to estimate parameters that lets us determine whether a particular gene belongs in the core or transient group within a robust statistical framework that gives us the means to adjust for false positives and multiple testing issues. (0.02 MB PDF) [file pcbi.1000626.s002.pdf]

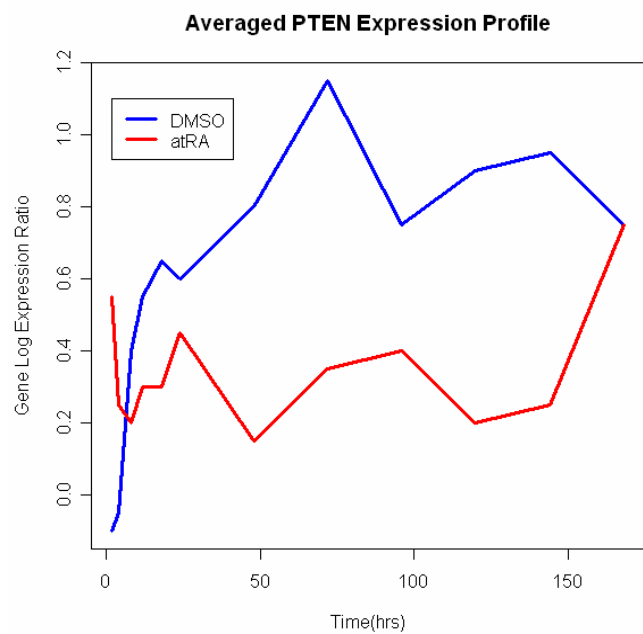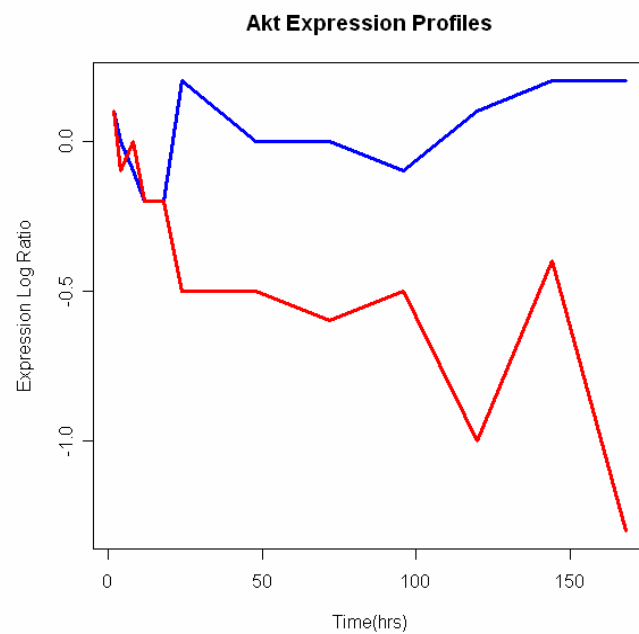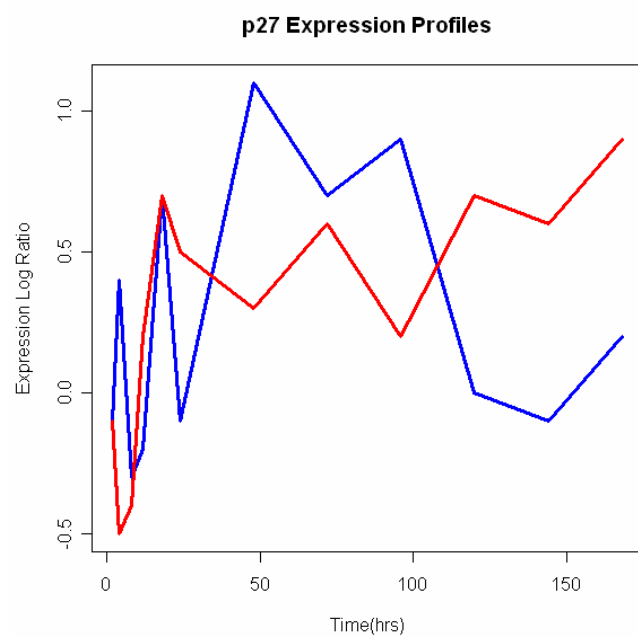

Supplement: Figure S2 — Expression profiles for some genes involved in DMSO-induced signaling. (0.03 MB PDF) [file pcbi.1000626.s003.pdf]

## Self-Organizing Map Clustering

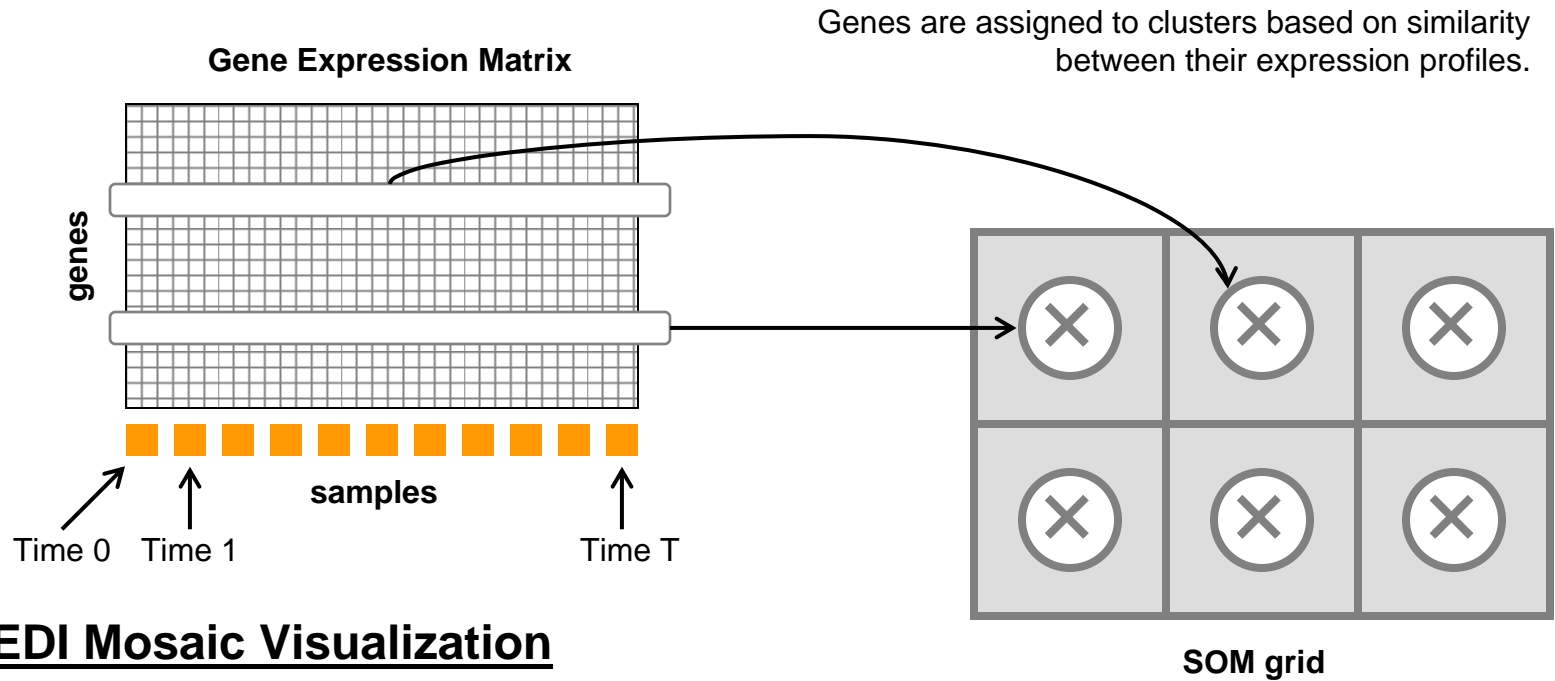

## GEDI Mosaic Visualization

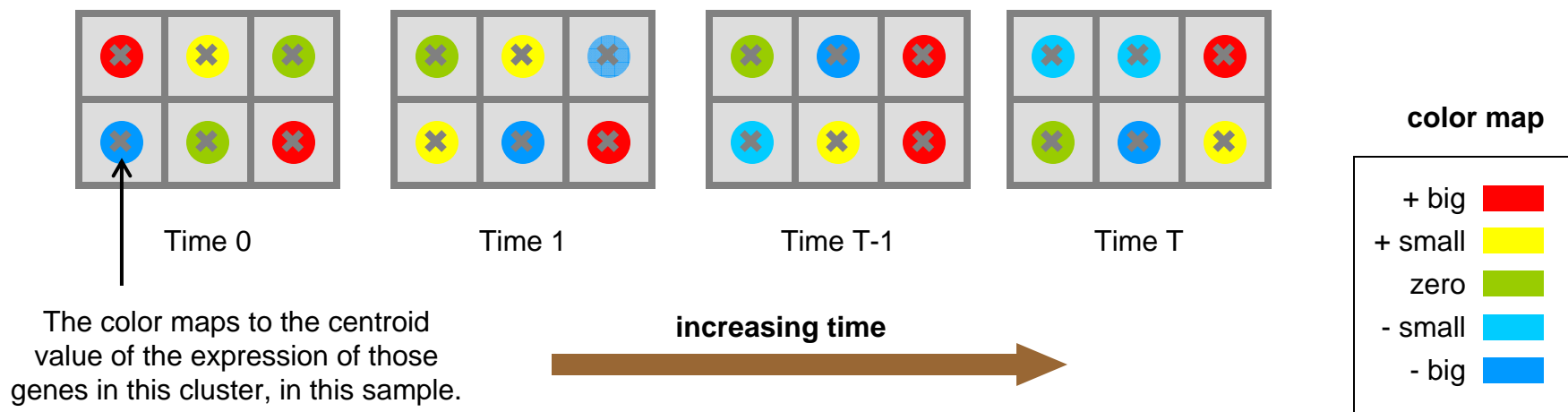

Supplement: Figure S6 — Cartoon describing how Figure 3 was constructed using GEDI software. (0.02 MB PDF) [file pcbi.1000626.s007.pdf]
