# Supplementary material for: Decomposition of Gene Expression State Space Trajectories
Source: PLoS Comput Biol. 2009 Dec 24;5(12):e1000626. doi: 10.1371/journal.pcbi.1000626 (PMC2791157; doi:10.1371/journal.pcbi.1000626)
Supplement: Figure S3 — Expression profiles for some genes involved in ATRA-induced signaling. (0.03 MB PDF) [file pcbi.1000626.s004.pdf]

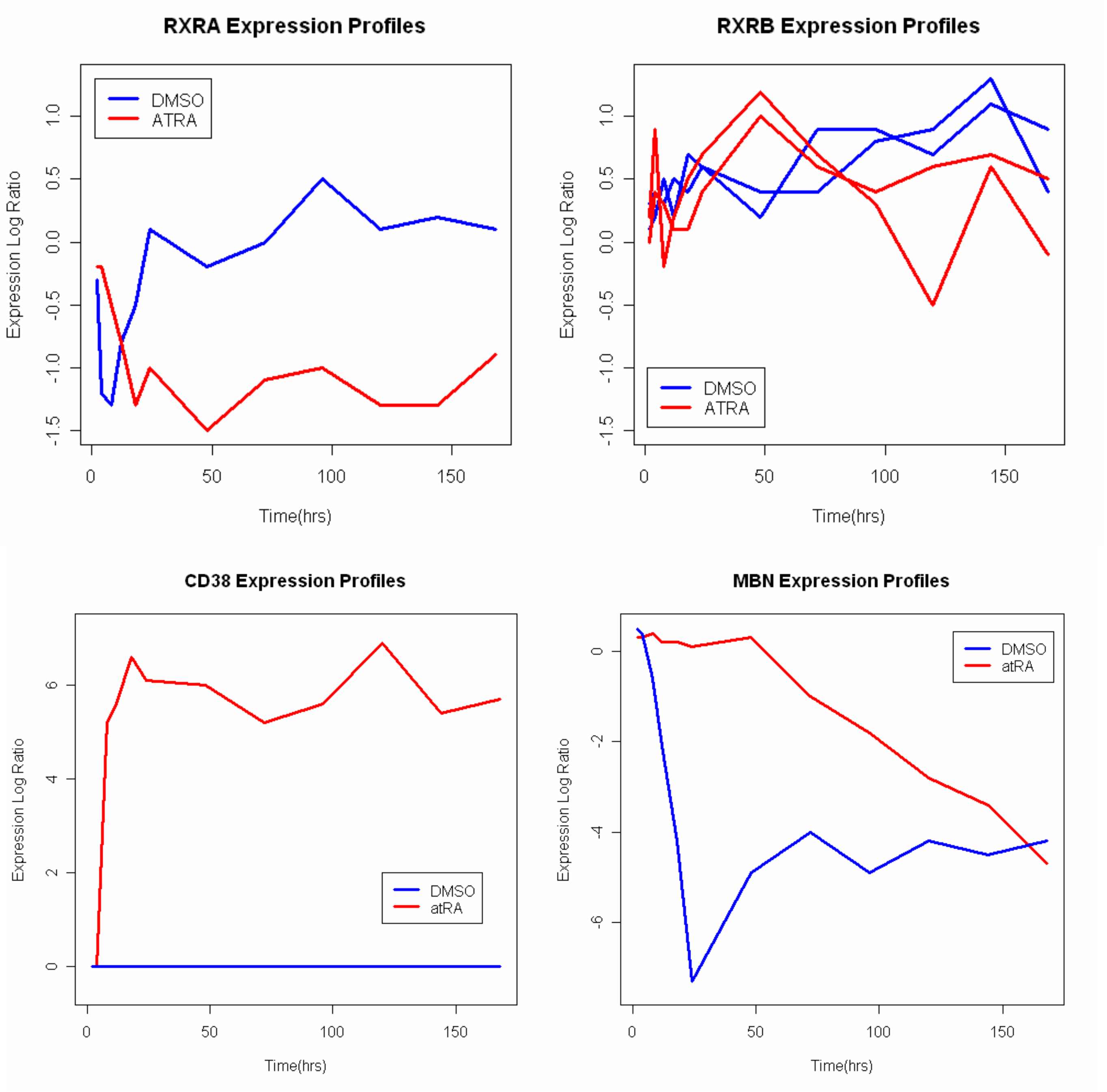

RXRA Expression Profiles
DMSO
ATRA
Expression Log Ratio
Time(hrs)
RXRB Expression Profiles
DMSO
ATRA
Expression Log Ratio
Time(hrs)
CD38 Expression Profiles
DMSO
atRA
Expression Log Ratio
Time(hrs)
MBN Expression Profiles
DMSO
atRA
Expression Log Ratio
Time(hrs)
