# Supplementary material for: Decomposition of Gene Expression State Space Trajectories
Source: PLoS Comput Biol. 2009 Dec 24;5(12):e1000626. doi: 10.1371/journal.pcbi.1000626 (PMC2791157; doi:10.1371/journal.pcbi.1000626)
Supplement: Figure S4 — Expression profiles for some genes known to participate in myeloid differentiation. (0.03 MB PDF) [file pcbi.1000626.s005.pdf]

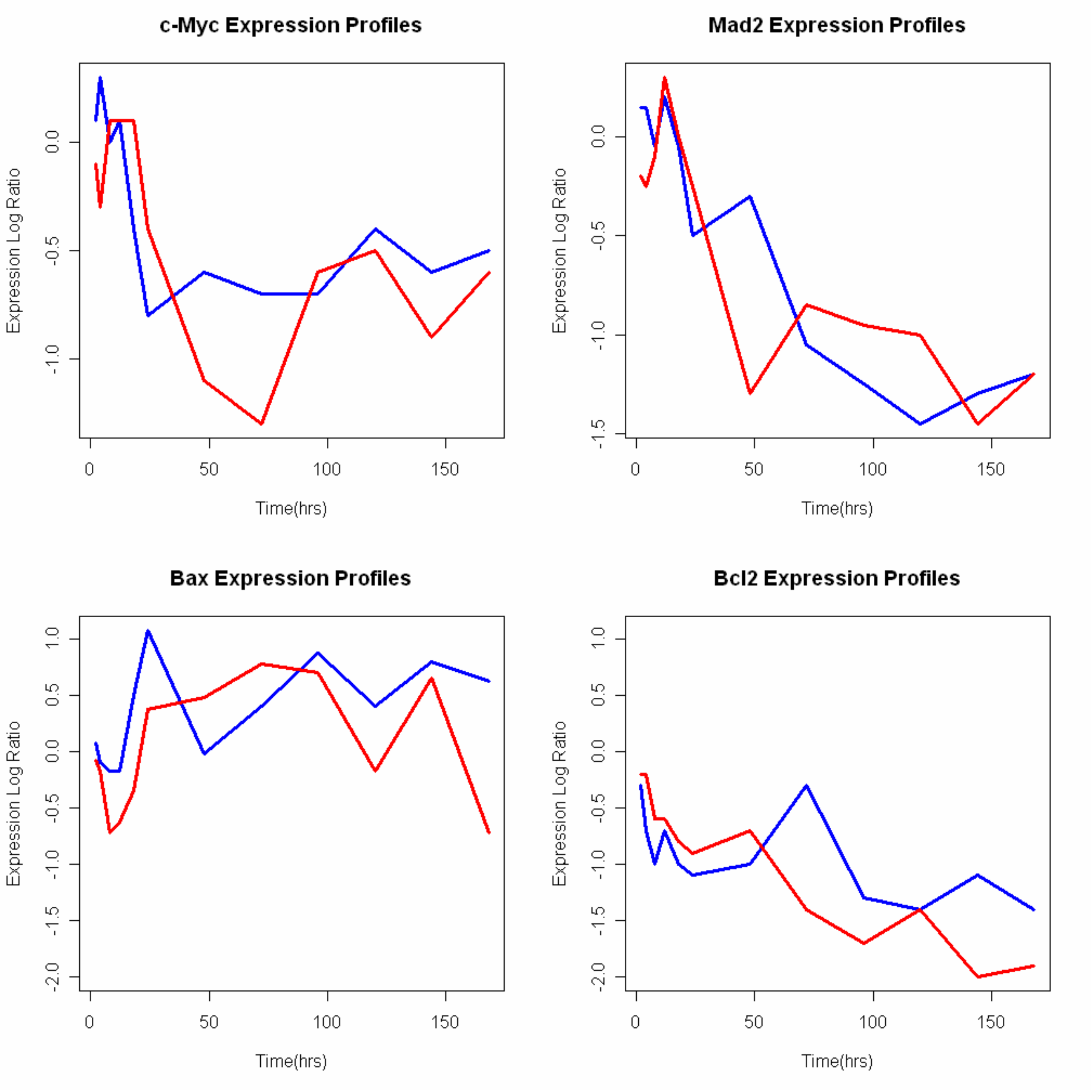
c-Myc Expression Profiles
Mad2 Expression Profiles
Bax Expression Profiles
Bcl2 Expression Profiles
Expression Log Ratio
Time(hrs)
0
50
100
150
0.0
-0.5
-1.0
-1.5
-2.0
0.5
1.0
