# Supplementary material for: Decomposition of Gene Expression State Space Trajectories
Source: PLoS Comput Biol. 2009 Dec 24;5(12):e1000626. doi: 10.1371/journal.pcbi.1000626 (PMC2791157; doi:10.1371/journal.pcbi.1000626)
Supplement: Figure S5 — Gene expression trajectories and their core and transient sub-components for the DMSO and ATRA-stimulated data. (0.02 MB PDF) [file pcbi.1000626.s006.pdf]

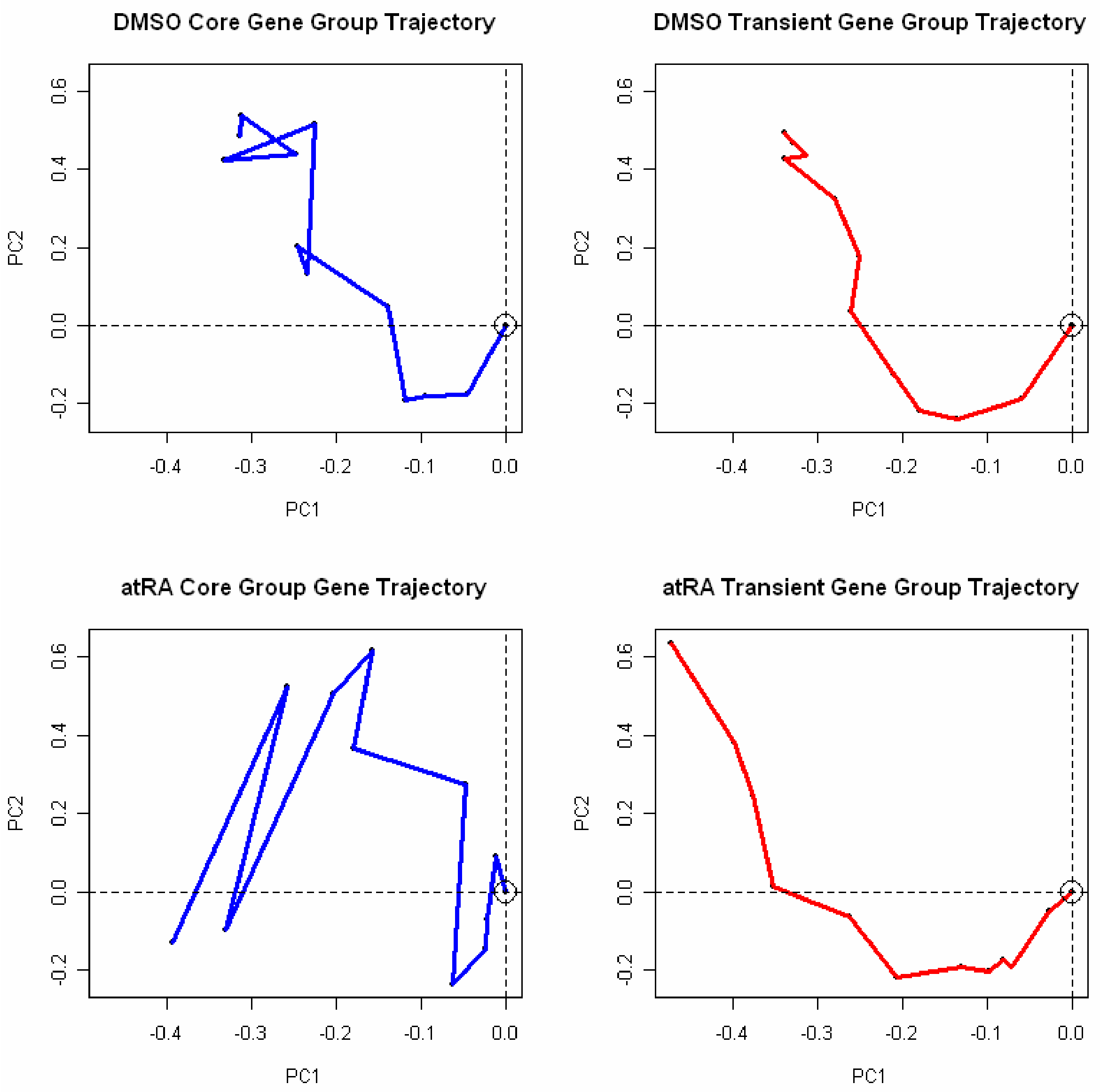
DMSO Core Gene Group Trajectory
DMSO Transient Gene Group Trajectory
atRA Core Group Gene Trajectory
atRA Transient Gene Group Trajectory
PC1
PC2
-0.4
-0.3
-0.2
-0.1
0.0
-0.2
0.2
0.4
0.6
