# Supplementary material for: Decomposition of Gene Expression State Space Trajectories
Source: PLoS Comput Biol. 2009 Dec 24;5(12):e1000626. doi: 10.1371/journal.pcbi.1000626 (PMC2791157; doi:10.1371/journal.pcbi.1000626)
Supplement: Table S1 — The core genes. Table S1 lists the 1428 genes that were placed in the core group. (0.06 MB PDF) [file pcbi.1000626.s008.pdf]

Supplemental Table 1: List of 1428 Core Genes

Affymetrix ID	GenBank Accession Number	Gene Symbol
39969_at	AA255502	HIST1H4C
37506_at	Z78308	PRPF40A
38533_s_at	J03925	ITGAM
40775_at	AL021786	ITM2A
31670_s_at	U81554	CAMK2G
34278_at	L18960	EIF1AX
32588_s_at	X78992	ZFP36L2
40742_at	M16591	HCK
37985_at	L37747	LMNB1
36209_at	S78771	BRD2
40725_at	AF047438	GOSR1
36982_at	U30888	USP14
32186_at	M80244	SLC7A5
40617_at	AC004381	THUMPD1
307_at	J03600	ALOX5
33389_at	U23942	CYP51A1
39158_at	AB021663	ATF5
663_at	L18960	EIF1AX
32706_at	X89887	HIRA
36671_at	M27396	ASNS
36857_at	AF084513	RAD1
41572_r_at	X75042	REL
32921_at	X83300	SMA4
37485_at	D88308	SLC27A2
41750_at	D49489	PDIA6
41242_at	AB011004	UAP1
37796_at	AF053356	LRCH4
37918_at	M15395	ITGB2
38378_at	M37033	CD53
1336_s_at	X06318	PRKCB1
34337_s_at	AJ010014	MTF2
32855_at	L00352	LDLR
32802_at	AB011169	39513
37324_at	X01060	TFRC
40296_at	AL023653	CXorf9
36030_at	AL080214	HOM-TES-103
35249_at	AF102778	CCNE2
41219_at	AL050376	USP34
38397_at	U09196	POLD4
39167_r_at	D83174	SERPINH1
1191_s_at	AB003102	PSMD11
39372_at	W26480	FADS1
39179_at	Z26248	PRG2
36858_at	D25218	RRS1
35007_at	AC004940	TWISTNB
32112_s_at	AW015732	AIM1
33816_at	AF020267	MYO9B
33283_at	AF106941	ARRB2
34183_at	AL080169	CCDC69
36629_at	AI635895	TSC22D3

Supplemental Table 1: List of 1428 Core Genes

1998_i_at	U19599	BAX
2002_s_at	U27467	BCL2A1
39330_s_at	M95178	ACTN1
241_g_at	M64231	SRM
37535_at	M27691	CREB1
36203_at	X16277	ODC1
31536_at	AB020693	RTN4
40424_at	AI017935	PROSC
33369_at	AI535653	SC4MOL
35985_at	AB023137	PALM2-AKAP2
40456_at	AL049963	SLC39A8
34172_s_at	M99578	SFRS17A
40521_at	AL050259	RGL2
1856_at	X75042	REL
1008_f_at	U50648	EIF2AK2
41819_at	U93049	FYB
35934_at	L19161	EIF2S3
32451_at	L35848	MS4A3
41683_i_at	U50708	BCKDHB
1836_at	D50310	CCNI
40490_at	U41387	DDX21
36899_at	M97287	SATB1
38671_at	AB014520	PLXND1
37037_at	M24486	P4HA1
32321_at	M16714	HLA-E
40399_r_at	AI743406	MEOX2
34223_at	M59818	CSF3R
35999_r_at	AB018324	SNF1LK2
38303_at	AB001523	TMEM1
33936_at	L23116	GALC
36197_at	Y08374	CHI3L1
596_s_at	M59820	CSF3R
41193_at	AB013382	DUSP6
34563_at	D26361	KIF14
1725_s_at	HG884-HT884	NA
38010_at	AF002697	BNIP3
41322_s_at	AI816034	NOLA2
39799_at	M94856	FABP5
38805_at	X89750	TGIF1
31867_at	AF052174	NA
673_at	J04031	MTHFD1
33294_at	D29958	EXOSC7
40463_at	U72395	TNPO1
33855_at	M96995	GRB2
35018_at	U61538	CHP
39566_at	X70297	CHRNA7
40719_at	AL022398	TRAF3IP3
40721_g_at	AL022398	TRAF3IP3
41047_at	AI885170	C9orf16
35779_at	AJ133421	VPS45
40923_at	AA290994	SLC36A1

Supplemental Table 1: List of 1428 Core Genes

794_at	X62055	PTPN6
32548_at	L24804	PTGES3
38435_at	U25182	PRDX4
41465_at	AJ236885	ZNF148
41223_at	M22760	COX5A
1081_at	M33764	ODC1
41691_at	AB018337	UBXD7
1602_at	L33881	PRKCI
40036_at	AF035940	MAGOH
40464_g_at	U72395	TNPO1
35295_g_at	M25077	TROVE2
39423_f_at	AJ000644	SPOP
39748_at	AL050021	SLC7A1
34741_at	U18422	TFDP2
35303_at	U96876	INSIG1
36190_at	M63256	CDR2
39839_at	M24069	CSDA
41591_at	AI652978	WDR68
36813_at	U96131	TRIP13
40495_at	AA306076	ORAI2
41246_at	AL040655	SERPINE2
41850_s_at	U63825	CCDC85B
37095_r_at	M84562	FPRL1
32113_at	U83115	AIM1
34206_at	AB018325	CENTD2
32737_at	M64595	RAC2
39049_at	AJ243937	GPSM3
39424_at	U70321	TNFRSF14
40569_at	M58297	MZF1
41252_s_at	W28614	PLEKHB2
1693_s_at	D11139	TIMP1
1217_g_at	X07109	PRKCB1
41720_r_at	AF009767	FADS1
34279_at	AL050141	NBPF10
39328_at	M11058	HMGCR
32803_at	AF104398	CNIH
32720_at	AA151716	ATG12
37282_at	AJ000186	MAD2L1
39353_at	U07550	HSPE1
41485_at	X02152	LDHA
41713_at	U09848	ZKSCAN1
35839_at	D78130	SQLE
1603_g_at	L33881	PRKCI
36561_at	X73424	PCCB
40441_g_at	AL080119	SERBP1
41133_at	U32519	G3BP1
442_at	X15187	HSP90B1
32047_at	U91985	DFFA
37174_at	D14660	MRPL19
2036_s_at	M59040	CD44
34912_at	AF052941	DAPK2

Supplemental Table 1: List of 1428 Core Genes

33804_at	U43522	PTK2B
34709_r_at	Z75331	STAG2
36870_at	AB018347	VPS8
40098_at	AF001434	EHD1
39062_at	AL008726	CTSA
32257_f_at	AF003001	TERF1
34318_at	AJ005896	PRAF2
34408_at	AF004222	RTN2
35824_at	AJ223321	ZNF238
40219_at	AI796944	HEXIM1
1146_at	HG3477-HT3670	NA
993_at	X54637	TYK2
957_at	HG2059-HT2114	NA
910_at	M15205	TK1
37975_at	X04011	CYBB
34777_at	D14874	ADM
40780_at	AF016507	CTBP2
1272_at	L19161	EIF2S3
35136_at	AL031387	NXT2
39041_at	Y00978	DLAT
36135_at	U86602	EBNA1BP2
40587_s_at	AF054186	EEF1E1
41510_s_at	L15189	HSPA9
36685_at	W63793	AMD1
38424_at	AB018290	FAM62A
33307_at	AL022316	CTA-126B4.3
39756_g_at	Z93930	XBP1
40151_s_at	Z48054	PEX5
1909_at	M14745	BCL2
1473_s_at	U22376	MYB
31443_at	S76346	RUNX1
35419_g_at	J04178	HEXA
34481_at	AF030227	VAV1
38216_at	L40411	JMJD1C
38895_i_at	X77094	NCF4
38945_at	X78710	MTF1
39280_at	U80744	TNRC5
41622_r_at	X78924	ZNF266
32740_at	AB023158	RAB11FIP2
35199_at	AB023199	WDR37
40045_g_at	AF009425	C18orf1
35341_at	U90547	TRIM38
36626_at	X87176	HSD17B4
39525_at	AL120687	PLEKHB2
2062_at	L19182	IGFBP7
1532_g_at	U50535	PFAAP5
1456_s_at	M63838	IFI16
362_at	Z15108	PRKCZ
364_s_at	Z16411	PLCB3
38831_f_at	AF053356	GNB2
1997_s_at	U19599	BAX

Supplemental Table 1: List of 1428 Core Genes

41760_at	AA978033	COX7A2
35818_at	U52111	CYCS
37263_at	U55206	GGH
41085_at	AF025840	POLE2
34286_at	AB020623	BCAS2
38676_at	AA059408	STCH
35364_at	U50939	APPBP1
34400_at	AI540957	UQCRQ
36992_at	AI653621	TXN
36012_at	Y09631	C13orf24
38260_at	AL050306	LAS1L
36191_at	M62810	TFAM
38076_at	X69907	ATP5G1
39088_at	Y18007	TMEM147
860_at	U03911	MSH2
37520_at	AJ006591	ZNF330
33230_at	AJ131186	PRPF19
35709_at	AF038172	RFK
39377_at	D87453	MRPS27
40861_at	D14812	MORF4L2
35255_at	AF098799	IPO7
37640_at	M31642	HPRT1
39385_at	M22324	ANPEP
33394_at	AW016836	DDX19A
34936_at	AB012130	SLC4A7
36317_at	U57057	CORO2A
37883_i_at	AI375033	C2orf27
39637_at	U14528	SLC26A2
36551_at	AL049382	C12orf29
39059_at	AF034544	DHCR7
40122_at	AF037448	SYNCRIP
37699_at	U29607	METAP2
1287_at	J03473	PARP1
34584_at	L75847	ZNF45
34046_at	Z83844	SH3BP1
37474_at	AF025441	OIP5
38894_g_at	AL008637	NCF4
38963_i_at	U12707	WAS
32116_at	AB002405	TMC6
32081_at	AB023166	CIT
34178_at	AI884738	ZBTB22
33254_at	AF008915	EVI5
36028_at	U45285	TCIRG1
36814_at	AB029032	KIAA1109
41767_r_at	AB020662	GOLGA8A
35842_at	AL049265	IL6ST
36093_at	AB014514	FLJ30092
40886_at	M29548	EEF1A1
40599_at	AL109669	IL16
40641_at	AF038362	BTAF1
1999_s_at	U19775	MAPK14

Supplemental Table 1: List of 1428 Core Genes

1842_at	HG2724-HT2820	NA
1556_at	U23946	RBM5
1165_at	D49950	IL18
817_at	U72209	YAF2
676_g_at	J04164	IFITM1
39436_at	AF079221	BNIP3L
36582_g_at	U09510	GARS
36631_at	D49396	PRDX3
39777_at	AF075587	MYCBP2
37668_at	M69039	C1QBP
34649_at	M14219	SSR1
33247_at	U86782	PSMD14
34716_at	AF067730	FUSIP1
40821_at	M61832	AHCY
36614_at	X87949	HSPA5
41488_at	AC002394	LYRM1
33214_at	Y11681	MRPS12
32124_at	AL030996	THOC2
32129_at	AL079314	ZNF364
32230_at	U39067	EIF3I
36206_at	L31801	SLC16A1
36592_at	S85655	PHB
40647_at	Z32684	XK
35631_at	U37689	POLR2H
36909_at	X62048	WEE1
34828_at	AL037557	POLR2I
36620_at	X02317	SOD1
37696_at	L06328	VDAC2
37694_at	D87685	PHF3
41829_at	AB018274	LARP1
1635_at	U07563	ABL1
260_at	M16447	QDPR
41403_at	AI032612	SNRPF
34773_at	AF038952	TBCA
36553_at	AA669799	ASMTL
32850_at	Z25535	NUP153
32853_at	AB018262	TOMM70A
33424_at	Y00281	RPN1
33932_at	X17644	GSPT1
34839_at	AB029027	PITRM1
38082_at	AB014550	SMCHD1
33123_at	L10379	THAP11
32734_at	L76703	PPP2R5E
33767_at	X15306	NEFH
36457_at	U10860	GMPS
33877_s_at	AB028990	EXOC7
35761_at	AL050073	AASDHPPT
37313_at	U80017	SMN1
37752_at	M15353	EIF4E
2086_s_at	D17517	TYRO3
1798_at	U41060	SLC39A6

Supplemental Table 1: List of 1428 Core Genes

36802_at	M23197	CD33
37415_at	AB018258	ATP10B
39298_at	AB022918	ST3GAL6
41400_at	K02581	TK1
41430_at	AB011113	WDR7
34226_at	U77129	MAP4K5
34757_at	AA595596	PARP2
36068_at	AF002210	CCS
36863_at	AF032862	HMMR
37915_at	AL080173	BAZ2B
38265_at	AI538172	RBBP6
38290_at	AF037195	RGS14
38976_at	D44497	CORO1A
39082_at	D00510	ANXA6
39420_at	S62138	FUS
40149_at	AL049924	SH2B1
40873_at	D86963	DVL3
32174_at	AF015926	SLC9A3R1
32254_at	AL050223	VAMP2
32833_at	M59287	CLK1
32834_r_at	AF013591	RIOK3
36675_r_at	J03191	PFN1
37026_at	U44975	KLF6
39133_at	AI525379	BLOC1S1
39891_at	AI246730	ZNF710
39873_at	X66360	PCTK2
40915_r_at	Y00272	CDC2
41598_at	AA890010	SEC22B
1386_at	M83738	PTPN9
1341_at	X52056	SPI1
1164_at	HG3344-HT3521	NA
603_at	M29960	NR2C1
558_at	M98776	KRT1
487_g_at	U60521	CASP9
349_g_at	D14678	KIFC1
32034_at	AF041259	ZNF217
39269_at	L07541	RFC3
32046_at	D10495	PRKCD
34811_at	U09813	ATP5G3
41735_at	AI808958	DENND3
36185_at	D32050	AARS
34460_at	AB014512	BZRAP1
41147_at	AF038186	ISCA1
32227_at	X17042	SRGN
37350_at	AL031177	PSMD10
31801_at	AI808712	TMF1
36898_r_at	X74331	PRIM2
34327_at	Z46606	HLTF
36962_at	U24105	COPA
34402_at	AB024327	STRAP
41606_at	AJ005940	DRG1

<u>**Supplemental Table 1: List of 1428 Core Genes**</u>

34879_at	AF007875	DPM1
35256_at	AL096737	SLC5A6
1990_g_at	U43746	BRCA2
193_at	U21858	TAF9
38846_at	AA768912	SSBP1
39806_at	AL050261	ASF1A
1721_g_at	U65410	MAD2L1
1161_at	J04988	HSP90AB1
39329_at	X15804	ACTN1
41733_at	AC003007	CTNNB1
36188_at	D32257	GTF3A
37403_at	X05908	ANXA1
38814_at	AF038954	ATP6V1G1
38462_at	U64028	NDUFA5
34651_at	M58525	COMT
35916_s_at	AA877215	HNRPA3
37844_at	AI263885	IL27RA
41632_at	D38550	E2F3
32695_at	Z97632	HTATSF1
36510_at	X16901	GTF2F2
38712_at	AL035291	C1orf9
40074_at	X16396	MTHFD2
34857_at	Z24724	ATP13A3
35300_at	X54326	EPRS
37663_at	X70649	DDX1
38797_at	D31887	SLC39A14
1248_at	U37689	POLR2H
262_at	M21154	AMD1
34718_at	X04434	IGF1R
40774_at	X74801	CCT3
34798_at	Z35491	BAG1
39884_g_at	AF091078	DIMT1L
34427_g_at	AF073485	MR1
35431_g_at	U78082	MED6
35905_s_at	U34995	GAPDH
35924_at	AI955897	INPP4A
36224_g_at	AI809970	SFPQ
37425_g_at	AB029343	CCHCR1
37826_at	AF020761	UBE2D1
38223_at	AB024057	TBC1D8
38547_at	Y00796	ITGAL
38558_at	M29273	MAG
32033_at	AL096780	CHKB
32079_at	AB014539	KIF13B
32133_at	AB011161	PIP5K1C
32632_g_at	J03060	GBA
32713_at	U51587	GOLGA1
33788_at	AB002390	ENTPD4
34690_at	U66616	SMARCC2
35166_at	D87343	DSCR3
36034_at	D87447	KIAA0258

Supplemental Table 1: List of 1428 Core Genes

36839_at	U77949	CDC6
37209_g_at	AJ001612	PSPHL
37544_at	X64318	NFIL3
39034_at	AL080122	CHMP2B
39339_at	AB018335	TMEM63A
40458_at	U43185	STAT5A
40783_s_at	L36151	PI4KA
39705_at	AB014600	SIN3B
40064_at	AB011121	TRAK2
40817_at	M96824	NUCB1
40877_s_at	AF041080	HERC2P2
41745_at	X57352	IFITM3
32145_at	X58141	ADD1
35363_at	AL080113	DDX17
34801_at	AB014610	USP52
34850_at	AB017644	UBE2E3
35328_at	AF055023	NF1
36958_at	X95735	ZYX
37047_at	AF002020	NPC1
40887_g_at	M29548	EEF1A1
39872_at	AL031588	GTSE1
40932_at	H18080	ZDHHC18
32577_s_at	L38933	PSMC3IP
1868_g_at	AF005775	CFLAR
1810_s_at	D10495	PRKCD
1503_at	X95152	BRCA2
1362_s_at	M84820	RXRB
1288_s_at	J04617	EEF1A1
1407_g_at	M21985	NR2C1
868_at	U13991	TAF10
845_at	U16031	STAT6
292_s_at	HG3484-HT3678	NA
187_at	U07349	MAP4K2
41855_at	AF030424	HAT1
32140_at	Y08110	SORL1
351_f_at	D28423	SFRS3
31869_at	AB014540	SWAP70
38374_at	AF050110	KLF10
1053_at	M87338	RFC2
34677_f_at	AJ012755	LOC220594
41220_at	AB023208	39700
33434_at	AF007551	BET1
861_g_at	U03911	MSH2
38148_at	D83702	CRY1
40634_at	M86667	NAP1L1
33370_r_at	U60205	SC4MOL
33429_at	AL050225	C18orf10
40279_at	D50911	VGLL4
40962_s_at	D26155	SMARCA2
1839_at	HG1112-HT1112	NA
549_at	S80343	RARS

Supplemental Table 1: List of 1428 Core Genes

34763_at	AF020043	SMC3
39396_at	AF081281	LYPLA1
40866_at	AJ001258	NIPSNAP1
1448_at	D00762	PSMA3
604_at	L78833	BRCA1
34099_f_at	W26056	NAP1L1
37250_at	AB007191	MYCBP
38702_at	AF070640	POLE3
39731_at	Z23064	RBMX
37009_at	AL035079	CAT
40789_at	U54645	AK2
32194_at	M37197	CEBPZ
35759_at	AF026166	CCT2
35836_at	AB019408	NUDC
37302_at	U30872	CENPF
37666_at	D29011	PSMB5
39561_at	AL008583	CBX6
1630_s_at	HG3730-HT4000	NA
357_at	AF000430	DNM1L
37825_at	M84443	GALK2
35614_at	AB012124	TCFL5
38703_at	AF005050	DNPEP
34848_at	X69141	FDFT1
38019_at	L37043	CSNK1E
38450_at	X69804	SSB
38804_at	U33286	CSE1L
34417_at	AL049437	DPY19L2P2
37143_s_at	AB002359	PFAS
37884_f_at	AI375033	C2orf27
37935_at	AF016369	PRPF4
40133_s_at	W28944	GRHPR
40816_at	L07758	PWP1
34411_at	Y10387	PAPSS1
38394_at	D42047	GPD1L
32544_s_at	L12535	RSU1
1924_at	U11791	CCNH
1154_at	J02645	EIF2S1
1138_at	L20859	SLC20A1
31957_r_at	M17886	RPLP1
32007_at	W29045	NA
34568_at	X82634	KRT33B
35083_at	M10119	FTL
35534_at	AB011086	GPRIN2
36437_s_at	AF064606	RANBP9
32315_at	U12202	RPS24
32318_s_at	AB004047	ACTB
32980_f_at	AI688098	HIST1H2BG
34033_s_at	U82278	LILRA2
36216_at	AF065485	SNX4
36784_at	J03071	CSHL1
36795_at	J03077	PSAP

Supplemental Table 1: List of 1428 Core Genes

36808_at	AF001846	PTPN22
37442_at	AL050378	DKFZp586I1420
37098_at	D38537	PPOX
37106_at	D89928	ZNF354A
37411_at	D30758	CENTB1
38158_at	D79987	ESPL1
38863_at	L07540	RFC5
39267_at	AF102265	PGM3
39968_at	U50136	LTC4S
41404_at	AJ010119	RPS6KA4
41443_at	U63127	PSD4
41614_at	AB014608	PARC
41643_at	X83301	SMA5
32673_at	U90543	BTN2A1
33269_at	AB003723	PIGQ
33296_at	AB020643	GLCE
33761_s_at	AB007962	FAM91A2
35170_at	AF044414	MAN2C1
36066_at	AB020635	KIAA0828
37556_at	M81637	GCA
38259_at	AB002559	STXBP2
38278_at	M62324	ARID5A
38668_at	AB011125	GPATCH8
39725_at	L10910	RBM39
39379_at	AL049397	C1orf121
40071_at	U03688	CYP1B1
40132_g_at	D89937	FSTL1
41222_at	AF067575	STAT6
41231_f_at	X13546	HMGN2
32208_at	AB002353	KIAA0355
32237_at	D87454	KIAA0265
32259_at	AB002386	EZH1
33339_g_at	M97936	STAT1
32801_at	AB002315	KIAA0317
34365_at	AF042386	PPIE
36949_at	U29171	CSNK1D
36598_s_at	L36818	INPPL1
36996_at	U41635	OS9
37024_at	AF010312	LITAF
37410_at	AJ224358	MED22
38069_at	Z67743	CLCN7
39140_at	AL079292	DHX29
39864_at	D78134	CIRBP
39865_at	AI890903	KCTD7
40555_at	M31470	RHOQ
41588_at	AA022949	FGF18
32540_at	AI762547	PPP3CC
32599_at	AF013168	TSC1
33188_at	U37221	PPIL2
2044_s_at	M15400	RB1
1917_at	X03484	RAF1

Supplemental Table 1: List of 1428 Core Genes

1119_at	J05249	RPA2
1054_at	M87339	RFC4
940_g_at	D12625	NF1
904_s_at	L47276	TOP2A
329_s_at	HG2238-HT2321	NA
721_g_at	D87673	HSF4
467_at	U63717	OSTF1
359_at	Y10659	IL13RA1
282_at	L16782	MPHOSPH1
157_at	U65011	PRAME
34878_at	AB019987	SMC4
276_at	L08069	DNAJA1
665_at	L20321	NEK4
34648_at	Z12830	SSR1
32617_at	W74442	FANCI
39232_at	U09284	LIMS1
33863_at	U65785	HYOU1
39643_at	U94703	POLG2
41718_g_at	AC004770	FADS1
34723_at	U79270	COX11
32790_at	D59253	NCBP2
36122_at	X59417	PSMA6
38050_at	D79986	BCLAF1
40957_at	D63881	SUZ12
1877_g_at	HG1103-HT1103	NA
39600_at	AL080110	PAQR3
41060_at	M73812	CCNE1
32096_at	AC005546	TRMT1
40778_at	AF035555	HSD17B10
38732_at	X91788	CLNS1A
1313_at	D38048	PSMB7
981_at	X74794	MCM4
31608_g_at	AJ002428	VDAC1
38849_at	AF038966	SCAMP1
36017_at	AF055016	C13orf1
35699_at	AF046918	BUB1B
37966_at	AA187563	PARVB
39755_at	Z93930	XBP1
40419_at	X85116	STOM
36639_at	AF067853	ADSL
37334_at	U23803	HNRNPA0
38744_at	N95406	SHFM1
1644_at	U36764	EIF3I
33632_g_at	AF023612	TXNL4A
36502_at	AB020641	PFTK1
37244_at	AA746355	UCHL3
38675_at	M18465	SNRPC
40111_g_at	U49283	IDH3B
41146_at	J03473	PARP1
33819_at	X13794	LDHB
33912_at	Y13834	ZMPSTE24

Supplemental Table 1: List of 1428 Core Genes

34344_at	AF044195	IKBKAP
35323_at	U78525	EIF3B
36604_at	D83004	UBE2N
36658_at	D13643	DHCR24
37016_at	D13900	ECHS1
39844_at	AI806379	LSM12
35039_at	D87466	DCUN1D4
39658_at	AB007901	ZBTB24
40348_s_at	W25866	ANP32E
31883_at	AF025794	MTRR
34270_at	AJ238097	LSM5
38986_at	D83485	PDIA3
39693_at	N53547	TMEM109
41722_at	U40490	NNT
40852_at	AB025254	TDRD7
32241_at	AL050265	TARDBP
34349_at	AJ011779	SEC63
36128_at	L40397	TMED10
36602_at	D21064	PMPCA
37673_at	X96586	NSMAF
38385_at	S65738	DSTN
40576_f_at	D89678	HNRPDL
41320_s_at	U69609	LRRFIP1
32530_at	X56468	YWHAQ
630_at	L39874	DCTD
32883_at	X77744	ZNF160
33484_at	Y10571	RNF2
35413_s_at	M77172	ZNF22
40000_f_at	AB023203	VPS13A
40454_at	X87241	FAT
39367_at	AA522537	ELAC2
40859_at	AI561196	ZC3H14
31961_r_at	AF070579	NA
31584_at	X16064	TPT1
31673_s_at	X65784	NA
35017_f_at	M80470	HLA-G
36332_at	U40391	AANAT
33994_g_at	M22919	MYL6
35450_s_at	U77948	GTF2I
36279_at	AB011128	KIAA0556
34532_at	AF035318	CUGBP2
36692_at	AF052099	MTMR9
38181_at	X57766	MMP11
38603_at	U09367	ZNF136
37146_at	AB007864	ATG2A
37421_f_at	X17093	HLA-F
37424_at	AB029343	CCHCR1
37450_r_at	X04409	GNAS
37524_at	AB011421	STK17B
37855_at	M95767	CTBS
38893_at	AL008637	NCF4

Supplemental Table 1: List of 1428 Core Genes

38920_at	AF016582	CHEK1
39224_at	AB011152	CENTD1
39261_at	L16896	ZBTB48
40308_at	AI830496	ATAD2B
41669_at	D83776	ZCCHC11
41399_at	AB029034	PHF8
41621_i_at	X78924	ZNF266
41640_at	AL031427	YIPF1
41698_at	AL031685	SLC9A8
41702_r_at	U93869	POLR3F
34215_at	L03426	SFRS17A
34699_at	AL050105	CD2AP
33705_at	L20971	PDE4B
33774_at	X98172	CASP8
34292_at	X92475	TMEM187
34660_at	U64998	RNASE6
34713_at	AB002357	KIF3B
34761_r_at	U41766	ADAM9
35645_at	AL050148	SNX1
36076_g_at	AL037167	RABL4
36529_at	AI885381	TRAPPC6A
35209_at	AB018309	EPM2AIP1
37238_s_at	AF014118	PKMYT1
36901_at	L01042	TMF1
38710_at	AL096714	OTUB1
38649_at	AB023187	FNDC3A
39043_at	AF006084	ARPC1B
40453_s_at	U30826	SFRS5
39373_at	AF035284	FADS1
39403_at	AB014578	DNAJC13
39742_at	U59863	TANK
40068_at	U26648	STX5
40494_at	AF043733	DEDD
41137_at	AB007972	PPP1R12B
41239_r_at	M90696	CTSS
32202_at	U67322	RBCK1
32209_at	AF052151	FAM89B
32218_at	AF034176	NA
32245_at	AF014837	METTL3
33822_at	Z14229	NUMA1
33349_at	AL049378	TNPO1
33444_at	D30756	NBR1
33848_r_at	U10906	CDKN1B
34367_at	AF006043	PHGDH
34812_at	W26099	GORASP1
36115_at	L29217	CLK3
36940_at	D86970	MYO18A
36184_at	L06419	PLOD1
36211_at	D87461	BCL2L2
37033_s_at	X13710	GPX1
37763_at	AL031228	RXRB

Supplemental Table 1: List of 1428 Core Genes

39103_s_at	H98552	DHRS1
39130_at	AB018313	VPS39
40182_s_at	AF055027	CARM1
41264_at	AL050172	RBM4B
41274_at	AA908993	DKFZp667G2110
41282_s_at	AA194159	PEX10
41292_at	L22009	HNRPH1
33205_at	AF064804	SUPT3H
1612_s_at	X56681	JUND
1497_at	L04270	LTBR
1318_at	X74262	RBBP4
330_s_at	HG2259-HT2348	NA
594_s_at	M55265	CSNK2A1
507_s_at	U43189	ELF2
510_g_at	U44378	SMAD4
240_at	M64231	SRM
37902_at	L13278	CRYZ
38090_at	AL050371	PISD
38774_at	U77942	STX7
41579_s_at	AI952267	PRKCI
651_at	L07493	RPA3
37493_at	H04668	CSF2RB
32635_at	AB029036	TRIM33
36636_at	M12267	OAT
34684_at	L36140	RECQL
34736_at	M25753	CCNB1
35227_at	U72066	RBBP8
1659_s_at	D78132	RHEB
1446_at	D00760	PSMA2
36015_at	N30625	APITD1
36873_at	D16532	VLDLR
32780_at	AB018271	DST
39142_at	AJ001810	NUDT21
39162_at	AA156987	GTF2H5
32954_at	U79263	RIF1
34188_at	AF070606	ATP2B1
39730_at	X16416	ABL1
32745_at	AF034091	MRPL40
33456_at	U24166	MAPRE1
36100_at	AF022375	VEGFA
36633_at	AA114830	AKAP10
37728_r_at	X78669	RCN2
1809_at	AB003698	CDC7
958_s_at	HG2274-HT2370	NA
41393_at	AF003540	ZNF195
39020_at	U82938	SIVA1
33423_g_at	AF052155	SEC13
33879_at	U79528	OPRS1
33906_at	AB001740	SSSCA1
35812_at	AJ133769	TNPO3
36581_at	U09510	GARS

Supplemental Table 1: List of 1428 Core Genes

36684_at	M21154	AMD1
36687_at	N50520	COX7B
38405_at	U25165	FXR1
32615_at	J05032	DARS
960_g_at	HG2463-HT2559	NA
36235_at	W26334	NFE2L2
40758_at	X81788	ICT1
35991_at	AA917945	LSM6
36885_at	L28824	SYK
40509_at	J04058	ETFA
40788_at	U84371	AK2
40810_at	U66615	SMARCC1
32774_at	AI541050	NDUFB8
41776_at	U70660	ATOX1
34790_at	S70154	ACAT2
36688_at	U11313	SCP2
37358_at	AI039880	UBE2E1
38097_at	AF010313	EI24
38123_at	D14878	CDC123
40976_at	AF052432	KATNB1
32546_at	U59309	FH
1789_at	U65928	COPS5
1516_g_at	HG4074-HT4344	NA
584_s_at	M30938	XRCC5
229_at	M37197	CEBPZ
37508_f_at	AA675900	PRPF40A
33315_at	M29204	C2orf3
39710_at	U30521	C5orf13
40516_at	L19872	AHR
33881_at	AA977580	ACSL3
35793_at	AB014560	G3BP2
35848_at	AL049432	ZMIZ1
36187_at	M36717	RNH1
36189_at	U10323	ILF2
36195_at	U07681	IDH3A
36610_at	D21852	R3HDM1
1073_at	M81601	TCEA1
633_s_at	L40386	TFDP2
33666_at	M16342	HNRNPC
36489_at	D00860	PRPS1
36864_at	AJ001625	PEX3
39380_at	AB014597	ANKRD17
41128_at	AF070537	TMEM183A
41241_at	D84273	NARS
41162_at	Y13936	PPM1G
32841_at	U19765	CNBP
35750_at	AL049948	ZC3H15
35294_at	M25077	TROVE2
36599_at	M55905	ME2
38073_at	AB007858	RNMT
38821_at	AJ002030	PGRMC2

Supplemental Table 1: List of 1428 Core Genes

39086_g_at	AA768912	SSBP1
39110_at	X55733	EIF4B
40618_at	H15872	CDV3
41557_at	D29641	SKIV2L2
32536_at	Z37986	EBP
32595_at	U07231	GRSF1
1643_g_at	U35113	MTA1
1633_g_at	U77735	PIM2
1550_at	U19796	MRPL28
37828_at	AL050064	RSBN1
38505_at	AL050151	EIF2C2
33734_at	AL022398	C1orf107
39745_at	AB011139	OPA1
41201_at	AF000430	DNM1L
32846_s_at	D13629	KTN1
34814_at	AL041443	SAE2
36177_at	X78627	TSN
37697_s_at	L08666	VDAC2
37707_i_at	M81118	ADH5
31962_at	L06499	RPL37A
31596_f_at	L02326	LOC91316
31695_g_at	X82877	RSC1A1
32429_f_at	X68688	ZNF33B
33601_at	AF052145	ZNF804A
33656_at	D23661	RPL37
33683_at	D50525	TI-227H
34573_at	U14187	EFNA3
34593_g_at	M13932	RPS17
32272_at	K00558	TUBA1B
36403_s_at	AI434146	ZNF101
34005_at	X73079	PIGR
38872_at	U95044	ZNF230
39262_at	U79266	SAC3D1
40290_f_at	L13972	ST3GAL1
40676_at	U37139	ITGB3BP
40685_at	U10868	ALDH3B1
40724_at	Y14443	ZNF200
41034_s_at	U92315	SULT2B1
32036_i_at	AF001175	RPP14
32119_at	AL049423	NA
41100_at	AB023172	CARD8
41101_at	D87464	FIG4
41372_at	AB020638	KIAA0831
41381_at	AB002306	CHD9
41411_at	AI566877	NDUFAF1
41638_at	D38552	PPWD1
32045_at	AB002331	DIDO1
33251_at	AB018322	TMCC1
33281_at	D63485	IKBKE
34676_at	AB029022	CENTG2
34715_at	U74612	FOXM1

Supplemental Table 1: List of 1428 Core Genes

35233_r_at	AI056696	CETN3
35238_at	AB000509	TRAF5
35252_at	AB011100	KIAA0528
35697_at	L76259	PTS
37977_at	AI138834	DTX2
38636_at	AB003184	ISLR
38700_at	M33146	CSRP1
37943_at	AB002319	ZFYVE26
37964_at	W25793	PCGF3
38971_r_at	AJ011896	TNIP1
39081_at	AI547258	MT2A
39431_at	AJ132583	NPEPPS
40050_at	AF069747	CBFA2T2
40828_at	D63476	ARHGEF7
32161_at	W26406	LONP2
40869_at	U50839	RBM6
41152_f_at	T89651	LOC729362
32178_r_at	AJ011915	SNAP23
32197_at	AF070548	SLC25A11
33380_at	AB005754	POLS
33869_at	AL080218	STAT5B
33905_at	AF072242	MBD2
34887_at	N92548	RDX
35784_at	U64520	VAMP3
36129_at	AB007857	SGSM2
36591_at	X06956	TUBA4A
36651_at	X15525	ACP2
37296_at	L28997	ARL1
37306_at	D38549	CYFIP1
37676_at	AF056490	PDE8A
37759_at	U51240	LAPTM5
38120_at	U50928	PKD2
38447_at	U08438	ADRBK1
38478_at	U08377	SFRS8
38741_at	U70728	PSCD2
39105_at	Z46389	VASP
39153_r_at	U06632	COIL
40549_at	L04658	CDK5
41554_at	AF069291	OSGIN2
41565_at	AF034373	ATXN2L
41843_r_at	W28275	YIPF4
32578_at	AW005997	MLX
41525_at	N25429	HMG20B
32612_at	X04412	GSN
2021_s_at	M73812	CCNE1
1981_s_at	X60287	MAX
1833_at	M68520	CDK2
1673_at	M14764	NGFR
1494_f_at	M33318	CYP2A6
1370_at	M29696	IL7R
1326_at	U60519	CASP10

Supplemental Table 1: List of 1428 Core Genes

1327_s_at	U67156	MAP3K5
1308_g_at	D14533	XPA
1224_at	X66363	PCTK1
826_at	U50553	DDX3X
1127_at	L07597	RPS6KA1
1131_at	L11285	MAP2K2
1064_at	U02680	TWF1
980_at	AF002020	NPC1
920_at	HG846-HT846	NA
906_at	L78440	STAT4
706_at	HG4582-HT4987	NA
646_s_at	L29218	CLK2
344_s_at	D13146	CNP
327_f_at	HG1800-HT1823	NA
228_at	M35416	RALB
179_at	U38980	PMS2L11
143_s_at	U75309	TAF5
160044_g_at	NM_001098	ACO2
38984_at	AB007896	PREPL
40002_r_at	AI935442	VPS13A
31488_s_at	S81916	PGK1
40355_at	AJ006266	WDHD1
1310_at	D26599	PSMB2
798_at	X74330	PRIM1
224_at	S81439	KLF10
32849_at	D80000	SMC1A
32808_at	X07979	ITGB1
36163_at	L13761	DLD
652_g_at	L07493	RPA3
36535_at	U04209	MFAP1
37584_at	AJ007669	FANCG
37585_at	X13482	SNRPA1
37561_at	AL031778	NFYA
37610_at	AI765280	C7orf44
38689_at	AL021937	C22orf28
39790_at	M23115	ATP2A2
40117_at	D84557	MCM6
32159_at	L00049	KRAS
41142_at	U62961	OXCT1
41778_at	U53347	SLC1A5
1953_at	AF024710	VEGFA
1544_at	U39817	BLM
33631_at	AF023612	TXNL4A
32336_at	X05236	ALDOA
41010_at	Y17829	HOMER1
41436_at	AJ224901	ZMYM2
36565_at	X98253	RNF113A
38720_at	AF026292	CCT7
38981_at	AA203354	NDUFB3
39727_at	AF023917	DUSP11
40150_at	AA205857	SNRPD3

Supplemental Table 1: List of 1428 Core Genes

35823_at	M63573	PPIB
36102_at	AF038962	VDAC3
36099_at	M69040	SFRS1
37000_at	AL035304	BRP44
39169_at	AF054184	SEC61G
32564_at	AA083129	SEC61B
2035_s_at	M55914	ENO1
452_at	U66615	SMARCC1
41407_at	L03411	RDBP
33710_at	U72515	MBOAT5
33795_at	AB006630	TCF20
35140_at	R59697	CDK8
35232_f_at	AI056696	CETN3
36554_at	Y15521	ASMTL
41732_at	AA310786	BOLA2
34340_at	AA173896	CYB5B
37029_at	X83218	ATP5O
37359_at	D14658	SPCS2
37739_at	M86737	SSRP1
38413_at	D15057	DAD1
39108_at	U22526	LSS
39113_at	AI262789	PDIA4
41248_at	AB014589	CSTF2T
32584_at	D38047	PSMD8
1449_at	D00763	PSMA4
33658_at	S54641	ZNF124
38155_at	U92538	ORC5L
41667_s_at	AJ006068	TGDS
31847_at	X59131	USPL1
41646_at	AA576724	TAOK3
32044_at	D13635	UBE3C
32086_at	U66561	ZNF184
38256_s_at	W21827	NGDN
38667_at	AA189161	GLOD4
40417_at	D43950	CCT5
32236_at	AF032456	UBE2G2
32766_at	Z83840	XRCC6
32785_at	D50929	EIF3A
36107_at	AA845575	ATP5J
36145_at	U51586	PUF60
37389_at	U51678	C11orf58
38410_at	X72964	CETN2
38765_at	AB028449	DICER1
39921_at	AI526089	COX5B
40250_at	U55766	KRR1
41574_at	Y09703	PNN
1450_g_at	D00763	PSMA4
37882_at	X63468	GTF2E1
40754_at	Z30093	GTF2H3
36500_at	AF027974	NSDHL
39390_at	AF052123	NUP133

Supplemental Table 1: List of 1428 Core Genes

40881_at	X64330	ACLY
41122_at	AB011173	AOF2
40868_at	AA442799	THUMPD1
41746_at	Z83840	NHP2L1
33422_at	AF052155	SEC13
34394_at	AB018327	ADNP
36201_at	D13315	GLO1
36588_at	AB018353	UNC84A
40984_at	W28255	76P
1640_at	U17714	ST13
41099_at	X84740	LIG3
34706_at	AB011090	MGA
35229_at	L39211	CPT1A
37555_at	X95263	PWP2
32851_at	AF036956	CUGBP2
33893_r_at	AB007939	CEP170
37044_at	D49490	PDIA5
38011_at	AB006572	C19orf2
39152_f_at	U06632	COIL
31923_f_at	U60269	METTL7A
35547_at	AF058056	SLC16A7
32350_at	AB026118	MALT1
35859_f_at	U38979	PMS2L3
35866_at	X78926	ZNF268
37468_at	AF058925	JAK2
38592_s_at	AI828210	NA
39251_at	M74091	CCNC
41437_at	AL080118	C14orf109
36071_at	AF070633	IPO9
37901_at	Y08991	PIK3R4
38660_at	F27891	COX6A2
40839_at	AL080177	UBL3
34316_at	W52024	RPS15A
35348_at	AF022116	PRKAB1
39096_at	AB028942	SON
40952_at	AA257983	LOC730092
1163_at	HG3254-HT3431	NA
520_at	U07358	MAP3K12
230_s_at	M54914	FSHB
37218_at	D64110	BTG3
1211_s_at	U84388	CRADD
1178_at	HG2846-HT2983	NA
398_at	X98743	DDX18
41474_at	Y08319	KIF2A
37453_at	AJ006267	CLPX
37171_at	X67155	KIF23
41356_at	W27619	BCL11A
32638_s_at	AI610467	SMG1
40472_at	AF007155	AGPAT7
40105_at	M65131	MUT
40893_at	AF058953	SUCLA2

Supplemental Table 1: List of 1428 Core Genes

34253_at	D83781	NUP160
34659_at	AB018334	NUP155
834_at	U40462	IKZF1
38511_at	AL109722	ZNF529
31786_at	AF051321	KHDRBS3
33278_at	AC004381	ACSM3
35662_at	U20536	CASP6
40461_at	AB007855	ZHX3
31623_f_at	K01383	MT1A
32065_at	S68134	CREM
32627_at	AF039023	RANBP6
37197_s_at	AL050006	DYNC2LI1
38286_at	AB028994	AMOT
40113_at	D87119	TRIB2
41761_at	M96954	TIAL1
33421_s_at	AB016247	SC5DL
38470_i_at	D86981	APPBP2
39905_i_at	AA402332	ARFGAP3
1876_at	HG1103-HT1103	NA
1264_at	M25393	PTPN2
579_at	M95724	CENPC1
1245_i_at	U25975	PAK2
39324_at	AL050078	PGAP1
40487_at	W26634	TRIM44
242_at	M64571	MAP4
32955_at	AL021546	TRIAP1
41029_at	U44799	U1SNRNPBP
34717_s_at	AF047448	FUSIP1
40440_at	AL080119	SERBP1
1775_at	L24559	POLA2
40727_at	AL080090	ANAPC10
34199_at	AJ131244	SEC24A
32676_at	M93405	ALDH6A1
38684_at	AJ010953	ATP2C1
1572_s_at	M10051	INSR
131_at	X83928	TAF11
37873_g_at	AF072468	JRK
32087_at	M65217	HSF2
1565_s_at	M96995	GRB2
109_at	Z97074	RABEPK
34517_at	L25798	HMGCS1
41363_at	AF027150	SIP1
33299_at	M37712	NA
33796_at	U73960	ARL4A
38275_at	AF054996	IMP4
34341_at	U00238	PPAT
31597_r_at	L36055	EIF4EBP1
32398_s_at	Z75190	LRP8
36427_at	W27129	ANKRD40
33480_at	X15393	MLN
39306_at	AF052514	PRSS16

Supplemental Table 1: List of 1428 Core Genes

41062_at	AA037278	PCGF1
41709_at	AF034803	PPFIBP2
35217_at	AL049404	MFAP3
36476_at	AF016270	BRD8
37269_at	D38496	LZTR1
40090_at	AI797997	WBSCR22
39077_at	U41843	DRAP1
40142_at	Z48570	SPA17
40439_at	AF047469	ASNA1
34328_s_at	L34673	HLTF
37380_at	X59268	GTF2B
39146_at	U72936	ATRX
39908_at	AF069735	TAF6L
32539_at	U51205	COPS8
842_at	U48251	ZMYND8
160042_s_at	X58431	HOXB6
37280_at	U59912	SMAD1
34321_i_at	D87120	FAM3C
38136_at	L76937	WRN
36920_at	U46024	MTM1
35773_i_at	AA527880	NDUFB7
35067_at	AI925946	AGGF1
38150_at	U22233	MTAP
39299_at	AB023188	FASTKD2
32167_at	AL050216	PMPCB
36956_at	L20852	SLC20A2
37678_at	U23070	BAMBI
39546_s_at	AA191426	NOL7
38563_at	AF039652	RNASEH1
35250_at	AL031670	RNF24
40517_at	AB002370	KIAA0372
34813_at	AL079283	EIF1AX
36204_at	Y00815	PTPRF
992_at	X52221	ERCC2
32041_r_at	AB007892	CDC5L
31882_at	AJ001340	RRP9
32455_s_at	U88153	PELP1
34096_at	AB020719	CEP152
32338_at	AL049980	DKFZP564C152
37837_at	AB020670	ADNP2
40686_at	AI985272	NMB
41695_at	AB007874	ZBTB43
34234_f_at	AI688640	NKTR
35240_at	W28983	WDR73
32829_at	X97544	TIMM17A
35362_at	AB018342	MYO10
37382_at	N25117	RPS26
41328_s_at	AL096717	EML2
33134_at	AB011083	ADCY3
33180_at	U68111	PPP1R2
1325_at	U59423	SMAD1

Supplemental Table 1: List of 1428 Core Genes

37978_at	D78177	QPRT
39550_at	AB011156	GLT25D2
41111_at	U68418	BCAT2
39286_at	D64109	TOB2
40325_at	AB014460	NTHL1
34687_at	AF052167	MRS2L
36717_at	AJ224162	LIAS
37521_s_at	H82458	ZNF330
38491_at	U11732	ETV6
39687_at	AI524873	ATXN10
33850_at	W28892	MAP4
41277_at	AW021542	SAP18
455_at	U66618	SMARCD2
37495_at	U63336	PRR3
41459_at	M73047	TPP2
33149_at	U73524	CLP1
35050_at	X82068	GRIA3
37466_at	D84488	RAB7L1
39966_at	AF059274	CSPG5
1072_g_at	M77810	GATA2
33661_at	U66589	RPL5
36388_at	U47129	PTHR2
35432_at	AF074723	MED6
36769_at	X85134	RBBP5
37491_at	D90359	TAF1
41380_at	AF053003	DPH2
38622_at	W28953	MTG1
38346_at	U62767	DUS4L
39055_at	M32886	SRI
39701_at	AB006625	PEG3
34376_at	AB019517	PKIG
36158_at	AF086947	DCTN1
39862_at	AA528252	POL3S
1670_at	L23959	TFDP1
35938_at	M72393	PLA2G4A
41854_at	M13666	MYB
40545_at	AB018566	PROSC
31633_g_at	Z95118	LOC442240
34104_i_at	AI147237	IGHG3
34510_at	AF070552	CDT1
38192_at	AB014543	CLUAP1
40654_at	U88871	PEX7
38788_at	M82827	PML
37159_at	U79259	AHDC1
37792_s_at	Y15572	RAD51L3
35141_at	Z97029	RNASEH2A
38795_s_at	X56687	UBTF
41672_at	AF007128	FBXL14
33720_at	L48692	PNO1
41211_at	AB018308	RBM12
32215_i_at	AB020685	RHOBTB3

Supplemental Table 1: List of 1428 Core Genes

35091_at	AA706226	NRG2
35097_at	U93163	MAGEB2
35588_at	AB011414	ZNF443
33466_at	AF038182	C5orf30
38226_at	W27152	INTS10
41479_s_at	AF029670	RAD51C
41682_s_at	U50708	BCKDHB
31868_at	AF060798	STK16
40510_at	AB007917	HS2ST1
35345_at	X83618	HMGCS2
35799_at	AL080081	DNAJB9
37045_at	D87443	SNX19
38067_at	D86957	39699
40238_at	AI801872	GPRC5B
34883_at	D87451	RNF10
32703_at	Y13115	PLK4
40562_at	AF011499	GNA11
36020_at	U28831	LOC400986
39885_at	W87858	KIF2A
33035_at	AL021397	TIPRL
34449_at	U13022	CASP2
33342_at	AF039029	SNUPN
36704_at	AB030654	AP4S1
37052_at	D26135	DGKG
35190_at	AL031447	THAP3
35269_at	AF093420	HSPBP1
1947_g_at	M60614	WIT1
37057_s_at	U21092	TRAF3
35346_at	AB007856	FEM1B
40639_at	AL021683	SCO2
41559_at	AA434319	GRAMD1B
529_at	U15932	DUSP5
31614_at	U88048	NA
34628_at	Y09321	TAF4B
34731_at	D80007	PDCD11
1090_f_at	M64936	NA
31888_s_at	AF001294	PHLDA2
40848_g_at	AB018293	MICAL2
35293_at	J04137	TROVE2
40993_r_at	AF055993	SAP30
35291_at	AL050287	BAG2
1983_at	X68452	CCND2
36796_at	X52075	SPN
37132_at	AF084367	INVS
38869_at	AB028992	PLCH1
33280_r_at	X80062	ACSM3
36045_at	AJ223948	ASCC3
36120_at	X63657	FVT1
32856_at	AB020626	MICAL3
38453_at	X15606	ICAM2
33597_at	U09411	ZNF132

Supplemental Table 1: List of 1428 Core Genes

40624_at	U48250	OLIG2
40343_at	AJ005814	HOXA7
35673_at	U02082	ARHGEF5
31403_at	U39905	SLC18A1
31431_at	U12255	FCGRT
31432_g_at	U12255	FCGRT
31444_s_at	M62895	ANXA2P3
31497_at	U19142	GAGE1
31514_at	AF034970	DOK2
31523_f_at	Z80780	HIST1H2BE
31559_at	U26209	SLC13A2
31737_at	M20543	ACTA1
31934_at	Z48614	ADAM10
32021_at	AI560890	LEMD2
32405_at	AB014607	ACOT11
32474_at	X96744	PAX7
32490_at	AC005955	CEACAM4
33027_at	W27906	CTGLF1
34560_at	Z18954	S100A5
35116_at	X80821	ANKRD12
36360_at	AB007976	RRP15
36439_at	AL080134	ULK2
32888_at	X52213	LTK
33955_at	M65291	IL12A
33997_at	AL049449	NA
34000_r_at	X58398	LOC90925
34446_at	AL049701	RABGAP1L
36270_at	U04343	CD86
34955_at	AF071202	ABCC4
35458_r_at	U18237	ABCB10
35479_at	AJ242015	ADAM28
35868_at	M91211	AGER
35936_g_at	Y08683	CPT1B
36227_at	AF043129	IL7R
36262_at	Z12173	GNS
36736_f_at	Y10275	PSPH
38599_s_at	AD001530	FAM50A
37789_at	AF064094	TADA2L
38129_at	X78713	GK
38142_at	U38904	ZNF211
38531_at	AA428150	OCEL1
38859_at	AL080141	SEC31B
38919_at	AB023226	KIAA1009
39221_at	AF004231	LILRB2
39223_at	AL096749	C1orf175
39624_at	D89078	LTB4R
39926_at	U59913	SMAD5
40375_at	X63741	EGR3
39940_at	AL080094	RPESP
40312_at	AJ005670	DACH1
40320_at	AF000367	CDC14A

Supplemental Table 1: List of 1428 Core Genes

40701_at	U75362	USP13
41056_at	AL050198	PART1
41077_at	AB011115	LOC643641
32122_at	L31573	SUOX
41475_at	U91512	NINJ1
41620_at	AB018259	DOCK4
31795_at	AL022329	ADRBK2
31837_at	U62317	TMEM112B
32138_at	L07807	DNM1
32636_f_at	AB007881	SMG1
32668_at	AL080076	SSBP2
34669_at	X96717	TFE3
36081_s_at	AB004848	SETD4
36523_at	L06133	ATP7A
35222_at	S67970	ZNF75
35228_at	Y08682	CPT1B
35246_at	U18934	TYRO3
35706_at	N32859	NR1D2
36074_at	U12897	IPW
36455_at	L41162	COL9A3
36543_at	J02931	F3
37248_at	U83411	CPZ
36879_at	M63193	ECGF1
36894_at	AL031846	CBX7
37213_at	X90392	DNASE1L1
37905_r_at	X66436	GNL1
37953_s_at	U78181	ACCN2
39735_at	AF069987	NIT1
40154_at	AL096725	TTLL3
40814_at	L40586	IDS
40829_at	AB028960	WDTC1
32158_at	U53174	RAD9A
41195_at	U49957	LPP
33350_s_at	Z78315	WDR45
33352_at	X57985	HIST2H2BE
33372_at	AI189226	RAB31
33419_at	U83857	API5
33446_at	W26407	ABAT
34303_at	AL049949	C10orf56
35367_at	AB006780	LGALS3
35763_at	AB011112	NBEAL2
36207_at	D67029	SEC14L1
36644_at	D29963	CD151
36575_at	S59049	RGS1
36584_at	X07173	ITIH2
36674_at	J04130	CCL4
36680_at	M24895	AMY2B
36683_at	AI953789	MGP
37048_at	U58970	TOMM34
37317_at	L25107	PAFAH1B1
38064_at	X79882	MVP

Supplemental Table 1: List of 1428 Core Genes

38101_at	AB011151	ZCCHC14
38369_at	U70451	MYD88
38425_at	U49719	HMGCL
39540_at	AF000561	ZBTB7A
39829_at	AB016811	ARL4C
38466_at	X82153	CTSK
39204_at	AF090097	ANTXR1
39459_at	W28765	LOC552889
39899_at	AC005525	CADM4
40570_at	AF032885	FOXO1
40888_f_at	W28170	EEF1A1
39855_at	AC005787	FZR1
40167_s_at	AF038187	WSB2
40215_at	D50840	UGCG
40246_at	U13897	DLG1
40536_f_at	AI254524	EIF5B
41254_at	N48190	NA
41291_at	AC004528	C19orf6
41571_at	AA872560	REL
41825_at	W26652	PINK1
41500_at	AI761818	SKI
41550_at	AF091071	RER1
41596_s_at	U43573	NAGLU
32603_at	W27118	RPA2
32609_at	AI885852	HIST2H2AA4
33137_at	Y13622	LTBP4
33164_at	AJ132545	TESK2
2053_at	M34064	CDH2
1894_f_at	HG3236-HT3413	NA
2069_s_at	L23805	CTNNA1
1948_f_at	U31511	NOS2A
1919_at	X16316	VAV1
1557_at	U24152	PAK1
1738_at	M81933	CDC25A
1638_at	U11732	ETV6
1589_s_at	L42243	IFNAR2
1533_at	U63139	RAD50
1457_at	M64174	JAK1
1397_at	L32976	MAP3K11
1269_at	M61906	PIK3R1
1056_s_at	M90391	IL16
877_at	M27691	CREB1
879_at	M30818	MX2
815_at	U70987	DOK1
698_f_at	HG3635-HT3845	NA
664_at	L19593	IL8RB
617_at	M24902	ACPP
509_at	U44378	SMAD4
459_s_at	U68485	BIN1
180_at	S82470	LENG4
160028_s_at	X12949	RET

Supplemental Table 1: List of 1428 Core Genes

160025_at	X70340	TGFA
