# Supplementary material for: Decomposition of Gene Expression State Space Trajectories
Source: PLoS Comput Biol. 2009 Dec 24;5(12):e1000626. doi: 10.1371/journal.pcbi.1000626 (PMC2791157; doi:10.1371/journal.pcbi.1000626)
Supplement: Table S2 — The transient genes. Table S2 lists the 1462 genes that were placed in the transient group. (0.06 MB PDF) [file pcbi.1000626.s009.pdf]

Supplemental Table 2: List of 1462 Transient Genes

Affymetrix ID	GenBank Accession Number	Gene Symbol
31506_s_at	L12691	DEFA3
37066_at	X55668	PRTN3
38457_at	L21715	TNNI2
33098_at	U28694	CCR3
34091_s_at	M14144	VIM
37194_at	M68891	GATA2
39997_at	AF005664	CFP
37096_at	M34379	ELA2
34362_at	M55531	SLC2A5
38717_at	AL050159	METTL7A
37724_at	V00568	MYC
1427_g_at	D89077	SLA
1973_s_at	V00568	MYC
36897_at	AL022327	MLC1
38972_at	AF052169	KCTD12
41814_at	M29877	FUCA1
36661_s_at	X06882	CD14
37701_at	L13463	RGS2
41038_at	M32011	NCF2
33362_at	AF094521	CDC42EP3
33412_at	Z83844	LGALS1
41471_at	W72424	S100A9
38631_at	M92357	TNFAIP2
40159_r_at	M55067	NCF1
35315_at	X02544	ORM1
1426_at	D89077	SLA
34177_at	AF038660	B4GALT2
1110_at	M21624	TRD@
37357_at	D00723	GCSH
37328_at	X07743	PLEK
31409_at	U35459	SERPINB10
34445_at	AB007940	RABGAP1L
39994_at	D10925	CCR1
1780_at	M19722	FGR
239_at	M63138	CTSD
39028_at	U72761	RANBP5
34840_at	AI700633	SERINC5
33371_s_at	U59877	RAB31
41300_s_at	AA477898	ITM2B
39827_at	AA522530	DDIT4
37105_at	M16117	CTSG
32643_at	L07956	GBE1
39681_at	AF060568	ZBTB16
40310_at	AF051152	TLR2
36493_at	M33552	LSP1
36889_at	M33195	FCER1G
32193_at	AF030339	PLXNC1
32819_at	AJ223352	HIST1H2BK
2045_s_at	M16592	HCK
875_g_at	M26683	CCL2

Supplemental Table 2: List of 1462 Transient Genes

38514_at	M27749	IGLL1
33942_s_at	AF004563	STXBP1
38614_s_at	U77413	OGT
1794_at	M92287	CCND3
41308_at	U37408	CTBP1
1985_s_at	X73066	NME1
33781_s_at	AF075599	UBE2M
35615_at	D50914	BOP1
39056_at	X53793	PAICS
40091_at	U00115	BCL6
33387_at	AB007854	GAS7
36617_at	X77956	ID1
36984_f_at	X89214	HPR
32562_at	X72012	ENG
32529_at	X69910	CKAP4
1852_at	X02910	TNF
893_at	M91670	UBE2S
1052_s_at	M83667	CEBPD
36292_at	U07225	P2RY2
2042_s_at	M15024	MYB
31858_at	X07315	NUTF2
39073_at	AL038662	NME1
39582_at	AL050166	CYLD
38326_at	M69199	G0S2
38363_at	W60864	TYROBP
39345_at	AI525834	NPC2
35820_at	M76477	GM2A
37011_at	U49392	AIF1
32574_at	X59960	SMPD1
32587_at	U07802	ZFP36L2
679_at	J04990	CTSG
37179_at	S77763	NFE2
36105_at	M18728	CEACAM6
41188_at	W28186	LAPTM4B
32791_at	L19183	TMEM97
35372_r_at	M28130	IL8
1500_at	X51630	WT1
36217_at	Z35102	STK38
32675_at	D21878	BST1
38006_at	M37766	CD48
34308_at	U90551	HIST1H2AC
36103_at	D90144	CCL3
1814_at	D50683	TGFBR2
1117_at	L27943	CDA
33963_at	M96326	AZU1
32749_s_at	AL050396	FLNA
38233_at	AF093265	HOMER3
33791_at	Y15227	DLEU1
37949_at	J03798	SNRPD1
41468_at	M30894	TARP
38160_at	AF011333	LY75

Supplemental Table 2: List of 1462 Transient Genes

41096_at	AI126134	S100A8
31870_at	X14046	CD37
35193_at	AF060219	RCBTB2
36488_at	AB011542	MEGF9
36922_at	X59618	RRM2
37995_s_at	M67468	FMR1
39143_at	U08015	NFATC1
1592_at	J04088	TOP2A
1173_g_at	HG172-HT3924	NA
461_at	U70063	ASAH1
34375_at	M28225	CCL2
446_at	U89896	CSNK1G2
38250_at	D26488	WDR43
1530_g_at	U50534	FRY
41213_at	X67951	PRDX1
41600_at	U87954	PA2G4
1521_at	X17620	NME1
33253_at	D50919	TRIM14
37726_at	X06323	MRPL3
39135_at	AB018310	DIP
38418_at	X59798	CCND1
38763_at	L29254	SORD
36287_at	X83368	PIK3CG
36313_at	M55267	EVI2A
37099_at	AI806222	ALOX5AP
41409_at	AF044896	C1orf38
32731_at	AB018247	APBB3
34745_at	AF070570	RAPGEF2
36495_at	U21931	FBP1
40081_at	L26232	PLTP
41198_at	AF055008	GRN
38087_s_at	W72186	S100A4
41796_at	AB029015	PLCL2
1913_at	U47414	CCNG2
1558_g_at	U24152	PAK1
1815_g_at	D50683	TGFBR2
35807_at	M21186	CYBA
41719_i_at	AF009767	FADS1
37842_at	AF054589	MDFIC
35771_at	AF049460	DEAF1
34965_at	AF031824	CST7
31863_at	D80001	RRP1B
36613_at	U09585	IFRD2
1474_s_at	U22376	MYB
1196_at	D00591	RCC1
37927_at	X12654	RCC1
36597_at	D21262	NOLC1
39812_at	X79865	MRPL12
38998_g_at	X96924	SLC25A1
36159_s_at	U29185	PRNP
38429_at	U29344	FASN

Supplemental Table 2: List of 1462 Transient Genes

32916_at	X54134	PTPRE
37472_at	U60337	MANBA
33223_at	AB011133	MAST3
33324_s_at	D88357	CDC2
37994_at	X69962	FMR1
38666_at	M85169	PSCD1
40862_i_at	X15334	CKB
33448_at	AB000095	SPINT1
34836_at	U18420	RAB5C
35369_at	AB023154	DTX4
36180_s_at	X75346	MAPKAPK2
36634_at	U72649	BTG2
36938_at	U70063	ASAH1
37398_at	AA100961	PECAM1
37723_at	U47414	CCNG2
38072_at	AL031432	C1orf63
38391_at	M94345	CAPG
40607_at	U97105	DPYSL2
1916_s_at	V01512	FOS
1830_s_at	M38449	TGFB1
1797_at	U40343	CDKN2D
1294_at	L13852	UBE1L
925_at	J03909	IFI30
810_at	U64105	ARHGEF1
756_at	D26350	ITPR2
33530_at	M33326	CEACAM8
36933_at	D87953	NDRG1
35695_at	U67615	LYST
31855_at	U61374	SRPX
32843_s_at	M30448	FBL
36957_at	W22296	ZMYND8
41827_f_at	AI932613	LOC91316
38618_at	AC002073	PPP1R14BP1
41754_at	M92439	LRPPRC
32825_at	Y10805	PRMT1
1468_at	U12595	TRAP1
34291_at	U07424	FARSA
41225_at	AL049417	DUSP3
1936_s_at	HG3523-HT4899	NA
674_g_at	J04031	MTHFD1
32317_s_at	U34804	SULT1A3
32868_at	L10386	TGM3
34435_at	AB008775	AQP9
36229_at	U58917	IL17RA
36780_at	M25915	CLU
38138_at	AC004668	S100A11
40720_at	AL022398	TRAF3IP3
41048_at	D90070	PMAIP1
36053_at	AF041248	CDKN2C
36509_at	AL049998	PIK3C2A
37967_at	AF000424	LST1

Supplemental Table 2: List of 1462 Transient Genes

38354_at	X52560	CEBPB
39032_at	AJ222700	TSC22D1
39728_at	J03909	IFI30
39061_at	D28137	BST2
39348_at	X99209	PRMT2
32253_at	AB007927	RERE
34387_at	D86960	LPGAT1
35739_at	AB002369	MTMR3
35785_at	W28281	GABARAPL1
38393_at	D87434	KIAA0247
39134_at	AJ006973	TOM1
41549_s_at	AF091077	AP1S2
2009_at	U33284	PTK2B
1925_at	Z36714	CCNF
1803_at	X05360	CDC2
1795_g_at	M92287	CCND3
1150_at	HG620-HT620	NA
478_g_at	U51127	IRF5
289_at	L29277	STAT3
36979_at	M20681	SLC2A3
40619_at	M91670	UBE2S
33131_at	X70683	SOX4
33977_at	U67369	GFI1
39661_s_at	AF034102	SLC29A2
40329_at	AL031228	SLC39A7
33243_at	AF099935	TNFAIP8
1470_at	U21090	POLD2
831_at	U28042	DDX10
430_at	X00737	NP
32165_at	L41887	SFRS7
37036_at	AB002299	MDN1
38729_at	M88279	FKBP4
39825_at	U25147	SLC25A1
37615_at	D86962	GRB10
36650_at	D13639	CCND2
36161_at	M34175	AP2B1
41342_at	D38076	RANBP1
1373_at	M31523	TCF3
32434_at	D10522	MARCKS
39397_at	M64497	NR2F2
41259_at	AI553745	HSPC111
32275_at	X04470	SLPI
32367_at	AL049634	SIRPB1
34914_at	U94778	PSTPIP1
38149_at	D29642	ARHGAP25
40019_at	M60830	EVI2B
31804_f_at	X78283	SULT1A1
37200_at	J04162	FCGR3B
38686_at	X71490	ATP6V0D1
39075_at	AF040958	NEU1
39689_at	AI362017	CST3

Supplemental Table 2: List of 1462 Transient Genes

40432_at	AA522891	GNS
40448_at	M92843	ZFP36
41215_s_at	D13891	ID2
32769_at	AB023210	WDFY3
33385_g_at	U31346	CAST
33839_at	D26350	ITPR2
34311_at	X76648	GLRX
36179_at	U12779	MAPKAPK2
38798_s_at	AI741833	AP1G2
38837_at	W26226	TXNDC13
39923_at	AI935420	ARHGAP12
40925_at	AA554945	C7orf44
40960_at	M22921	B4GALT1
41491_s_at	AB028944	ATP11A
32606_at	AA135683	BASP1
1038_s_at	U19247	IFNGR1
210_at	M95678	PLCB2
175_s_at	U33053	PKN1
160029_at	X07109	PRKCB1
40095_at	J03037	CA2
35926_s_at	AF009005	LILRB1
32070_at	X97267	PTPRCAP
32173_at	X95384	HRSP12
36766_at	X55988	RNASE2
33799_at	U76248	SIAH2
35758_at	AB024301	RUVBL2
1750_at	AD000092	FARSA
41408_at	AF042169	SUPV3L1
40124_at	Y18418	RUVBL1
41490_at	Y00971	PRPS2
749_at	D78586	CAD
37686_s_at	Y09008	UNG
1942_s_at	U37022	CDK4
1306_at	D12686	EIF4G1
31528_f_at	Z83738	HIST1H2BM
31508_at	S73591	TXNIP
31510_s_at	Z48950	H3F3B
36347_f_at	Z98744	HIST1H2BN
36417_s_at	AF035295	ACAA1
32901_s_at	AC005192	IFRD1
36218_g_at	Z35102	STK38
40697_at	X51688	CCNA2
41396_at	AB006629	CLIP2
31802_at	D86979	KIAA0226
33227_at	AI984234	IL10RB
34752_at	AL080111	NEK7
36564_at	W27419	RNF19B
36856_at	W28743	ANKDD1A
37173_at	Z15005	CENPE
37272_at	X57206	ITPKB
39417_at	AB028951	CDC2L6

Supplemental Table 2: List of 1462 Transient Genes

35299_at	AB000409	MKNK1
35317_at	AB014579	MGEA5
40585_at	D25538	ADCY7
40244_s_at	AI743654	MPPE1
41249_at	AL031282	NADK
41594_at	M64174	JAK1
1943_at	X51688	CCNA2
1824_s_at	J05614	PCNA
1398_g_at	L32976	MAP3K11
1347_at	S78187	CDC25B
527_at	U14518	CENPA
318_at	D64142	H1FX
106_at	Z35278	RUNX3
41654_at	X02994	ADA
33284_at	M19507	MPO
38510_at	AL049435	NA
605_at	L78833	VAT1
34283_at	AL050125	XYLT1
36453_at	AB018254	KBTBD11
36322_at	AB012668	FUT7
40522_at	X59834	GLUL
34319_at	AA131149	S100P
35879_at	M77140	GAL
41058_g_at	AI760162	THEM2
38030_at	AB002330	SR140
38164_at	U57629	RPGR
40365_at	M63904	GNA15
35620_at	AF043250	TOMM40
36088_at	AJ006291	DSCR2
39357_at	U72514	EMG1
37700_at	X92106	BLMH
38811_at	D82348	ATIC
34998_at	AF059531	PRMT3
41670_at	R38263	D15Wsu75e
37991_at	L38961	STT3A
37632_s_at	X98260	ZRF1
38678_at	AA733050	SNRPE
40198_at	L06132	VDAC1
40982_at	AA926957	TSR1
33113_at	U65093	CITED2
1751_g_at	AD000092	FARSA
1126_s_at	L05424	CD44
32004_s_at	W32483	CDC2L2
40696_at	U50062	RIPK1
41084_at	AI659108	ZNF428
32142_at	U26424	STK3
35251_at	X51435	HIVEP1
36465_at	U51127	IRF5
36843_at	AB005666	SIPA1
38297_at	X98654	PITPNM1
39057_at	L04733	KLC1

Supplemental Table 2: List of 1462 Transient Genes

39388_at	AA902713	CAMK2G
40082_at	D10040	ACSL1
41747_s_at	U49020	MEF2A
33390_at	AA203487	CD68
33453_at	AI400326	PTPN18
33841_at	R48209	KIAA1539
34304_s_at	AL050290	SAT1
34409_at	AL080164	LRP10
34892_at	AF016266	TNFRSF10B
36136_at	AF010315	TP53I11
36152_at	X79354	GDI1
36578_at	U37547	BIRC2
37377_i_at	M13452	LMNA
38381_at	U32315	STX3
38810_at	AF039241	HDAC5
39530_at	L35240	PDLIM7
40203_at	AJ012375	EIF1
41258_at	N29665	NSUN5C
41809_at	AI656421	C7orf23
33121_g_at	AF045229	RGS10
1461_at	M69043	NFKBIA
946_at	D50663	DYNLT1
857_at	S87759	PPM1A
497_at	U32680	CLN3
464_s_at	U72882	IFI35
33813_at	AI813532	TNFRSF1B
894_g_at	M91670	UBE2S
1583_at	M32315	TNFRSF1B
41734_at	AB020677	DENND3
38359_at	Y12336	RASGRP2
37695_at	D79983	RNF144A
33753_at	AB014566	DAAM1
41126_at	AA978353	SLC1A4
32543_at	M84739	CALR
922_at	J02902	PPP2R1A
35681_r_at	AB011141	ZEB2
32611_at	X75252	PEBP1
35963_at	AI201243	PFDN6
41106_at	AF022797	KCNN4
1911_s_at	M60974	GADD45A
1100_at	L76191	IRAK1
41869_at	U78310	PES1
33815_at	J03626	UMPS
34765_at	D13645	KIAA0020
33837_at	AF069765	SRP72
39173_at	X56597	FBL
2012_s_at	U34994	PRKDC
39677_at	D80008	GINS1
40347_at	AA913812	ANP32E
33708_at	AF045584	SLC43A1
35731_at	X16983	ITGA4

Supplemental Table 2: List of 1462 Transient Genes

37945_at	U91316	ACOT7
33348_at	M80627	TCF12
38753_at	AF039022	XPOT
39175_at	D25328	PFKP
40537_at	AB018284	EIF5B
35008_at	AB002345	PER2
31838_at	U79274	C12orf24
33250_at	AL031228	WDR46
39355_at	X76388	ABCE1
41209_at	M15856	LPL
37698_at	X97335	AKAP1
38384_at	X54199	GART
266_s_at	L33930	CD24
31672_g_at	D82351	RBMS1
35132_at	X98411	MYO1F
35012_at	M81750	MNDA
35466_at	W73046	DNAJB12
37100_at	AJ008112	FMNL1
39319_at	U20158	LCP2
33228_g_at	AI984234	IL10RB
33263_at	X67098	ENOSF1
34749_at	U83461	SLC31A2
35974_at	U10485	LRMP
37177_at	Y00636	CD58
38287_at	AA808961	PSMB9
39340_at	M16424	HEXA
39708_at	L29277	STAT3
40120_at	X90999	HAGH
40415_at	X14813	ACAA1
40518_at	Y00062	PTPRC
40841_at	AF049910	TACC1
32775_r_at	AB006746	PLSCR1
32166_at	AB028950	TLN1
32182_at	AB023182	STK38L
32824_at	AF039704	TPP1
33367_s_at	D88674	AZIN1
33895_at	AL050373	SH3YL1
35302_at	AJ132712	NXF1
35783_at	H93123	VAMP3
36142_at	X79204	ATXN1
36607_at	Z99716	NAGA
37003_at	X62654	CD63
37025_at	AL120815	LITAF
37366_at	AL049969	PDLIM5
37679_at	Y10313	IFRD1
38110_at	AF000652	SDCBP
38445_at	Y09160	ARHGEF1
39176_f_at	M54994	CEL
40282_s_at	M84526	CFD
40568_at	L35249	ATP6V1B2
1976_s_at	X06292	FES

Supplemental Table 2: List of 1462 Transient Genes

1915_s_at	V01512	FOS
1844_s_at	L05624	MAP2K1
1134_at	L13738	TNK2
883_s_at	M54915	PIM1
333_s_at	HG2639-HT2735	NA
450_g_at	U66469	CGRRF1
419_at	X65550	MKI67
319_g_at	D64142	H1FX
160027_s_at	Y00285	IGF2R
37027_at	M80899	AHNAK
33097_at	U80982	CEBPE
36372_at	U51333	HK3
34785_at	AB028948	MED13L
32550_r_at	Y11525	CEBPA
36175_s_at	AL023584	HIVEP2
37310_at	X02419	PLAU
1529_at	U50534	FRY
37215_at	AF046798	PYGL
37920_at	U70370	PITX1
40147_at	U18009	VAT1
32800_at	U66306	RXRA
39971_at	M22637	LYL1
41199_s_at	W27050	SFPQ
1405_i_at	M21121	CCL5
1369_s_at	M28130	IL8
35983_at	AC004528	WDR18
38311_at	AF055012	TGIF2
40506_s_at	U75686	PABPC4
34380_at	AC004472	STOML2
895_at	L19686	MIF
39932_at	AI655015	NA
37050_r_at	AI130910	TOMM34
33135_at	U17566	SLC19A1
39638_at	S73885	TFAP4
39980_at	AB000449	VRK1
40756_at	AF081280	NPM3
31899_at	D14659	TTC35
35643_at	X76732	NUCB2
37281_at	D87071	FAM38A
39767_at	D13627	CCT8
40827_at	U04953	IARS
32205_at	AF072860	PRKRA
1388_g_at	J03258	VDR
40354_at	AB023421	HSPA4L
31821_at	AB011167	PPRC1
36460_at	AF008442	POLR1C
38687_at	AL050051	ANAPC13
38996_at	U15655	ERF
34882_at	Y12065	NOL5A
38036_at	L35035	RPIA
931_at	L08177	EBI2

Supplemental Table 2: List of 1462 Transient Genes

31697_s_at	J04755	FTHP1
34642_at	U28964	YWHAZ
32964_at	X81479	EMR1
36312_at	L40377	SERPINB8
37470_at	AF013249	LAIR1
37509_at	AF046059	CRLF3
31860_at	X51804	TMEM11
32091_at	AB007915	SLC25A44
32696_at	X59841	PBX3
33305_at	M93056	SERPINB1
33308_at	M15182	GUSB
33333_at	AB007863	PIP3-E
34192_at	AB011104	VPS13B
34735_at	U43195	ROCK1
35172_at	AF049891	TPST2
36062_at	AF062075	LPXN
37278_at	X92762	TAZ
38264_at	U74324	RABIF
38641_at	AJ133115	TSC22D4
39369_at	AB023152	MAN2B2
41202_s_at	AF000152	CTDSP2
41758_at	AL096879	TMEM184B
41784_at	AL080186	SFRS18
32195_at	AL049450	MSL-1
33916_at	AB023192	NISCH
32826_at	AJ133133	ENTPD1
33854_at	AA877795	ATP6V1D
34835_at	D87442	NCSTN
34827_at	AF045458	ULK1
34877_at	AL039831	JAK1
35329_at	AF091084	CYB5R1
35770_at	D16469	ATP6AP1
36151_at	U60644	PLD3
36170_at	D83198	ETHE1
36963_at	W28263	PGD
37021_at	X16832	CTSH
37298_at	AF044671	GABARAP
37346_at	M57567	ARF5
37406_at	X94232	MAPRE2
39099_at	X97064	SEC23A
39120_at	AA224832	MT1X
40193_at	X51956	ENO2
1906_at	HG511-HT511	NA
1696_at	D29013	POLB
1568_s_at	L42243	IFNAR2
1536_at	U77949	CDC6
1305_s_at	D12620	CYP4F3
903_at	L42373	PPP2R5A
286_at	L19779	HIST2H2AA3
201_s_at	S82297	B2M
37054_at	J04739	BPI

Supplemental Table 2: List of 1462 Transient Genes

33454_at	AF016903	AGRN
40164_at	X69550	ARHGDIA
37185_at	Y00630	SERPINB2
37186_s_at	U11863	ABP1
649_s_at	L06797	CXCR4
1515_at	HG4074-HT4344	NA
39253_s_at	M29893	RALA
835_at	U41745	PDAP1
35414_s_at	U77914	JAG1
39089_at	Y07604	NME4
1403_s_at	M21121	CCL5
35972_at	AA181196	RNASEH2B
36264_at	S75168	MATK
37445_at	AB015633	TMEM5
38728_at	D86978	NUP205
38794_at	X53390	UBTF
32102_at	AB018273	SACS
41749_at	U53003	C21orf33
36987_at	M94362	LMNB2
38473_at	M63180	TARS
38782_at	M95809	GTF2H1
41807_at	AL040137	NA
36926_at	X80692	MAPK6
38727_at	M23161	MCFD2
34171_at	AI867349	NCLN
37501_at	Y07707	NKRF
35180_at	AL050205	LARP4
36928_at	AJ011806	ZNF146
39418_at	AJ007398	RSL1D1
41250_at	U24169	JTV1
33943_at	L20941	FTH1
33944_at	S60099	APLP2
34433_at	AF035299	DOK1
38575_at	AF070528	MALT1
37147_at	AF020044	CLEC11A
38188_s_at	L28821	MAN2A2
38964_r_at	U12707	WAS
39230_at	AL022318	APOBEC3B
40041_at	AF017790	NDC80
40726_at	U37426	KIF11
41386_i_at	AB002344	JMJD3
41618_at	M91669	COL17A1
41634_at	D87445	KIAA0256
32104_i_at	U66063	CAMK2G
32135_at	U00968	SREBF1
32681_at	S68616	SLC9A1
33759_at	X04327	BPGM
35646_at	Z35093	SURF1
35655_at	AB002377	ANKRD28
36921_at	U02556	DYNLT3
37268_at	U43368	VEGFB

Supplemental Table 2: List of 1462 Transient Genes

38310_at	AB014591	CNOT3
38704_at	AB007934	MACF1
37961_at	U90907	PIK3R3
38266_at	W25798	RBBP6
38610_s_at	X14487	KRT10
39003_at	Z50022	PTTG1IP
40779_at	U59919	KIFAP3
39402_at	M15330	IL1B
40153_at	X57522	TAP1
40446_at	AL021366	PHF1
40505_at	AA883502	UBE2L6
41168_at	AF029750	TAPBP
32207_at	M64925	MPP1
33833_at	J05243	SPTAN1
33438_at	AL049981	WBP2
33867_s_at	X77494	RBMS1
34830_at	W25986	ECOP
35267_g_at	AL049288	BLCAP
34876_at	U65090	CPD
35336_at	AL021707	UNC84B
35767_at	AI565760	GABARAPL2
37294_at	X61123	BTG1
37384_at	D86995	PPM1F
37688_f_at	M31932	FCGR2A
38104_at	U78302	DECR1
38121_at	X59892	WARS
39150_at	U69559	RNF11
40235_at	L13738	TNK2
41288_at	AL036744	CALM1
1986_at	X74594	RBL2
1825_at	L33075	IQGAP1
1505_at	D00596	TYMS
1434_at	U92436	PTEN
1166_at	D78151	PSMD2
1243_at	U18300	DDB2
907_at	M13792	ADA
802_at	X84002	TAF12
653_at	L07540	RFC5
641_at	L76517	PSEN1
574_s_at	M87507	CASP1
404_at	X52425	IL4R
402_s_at	X69819	ICAM3
101_at	Y09305	DYRK4
167_at	U49436	EIF5
39649_at	X78817	ARHGAP4
40332_at	AF109134	OGFR
40567_at	AF081484	TUBA1B
812_at	U68111	PPP1R2
198_g_at	U29656	NME3
37674_at	Y00451	ALAS1
1519_at	J04102	ETS2

Supplemental Table 2: List of 1462 Transient Genes

32554_s_at	Y12781	TBL1X
40690_at	X54942	CKS2
1506_at	D11086	IL2RG
37827_r_at	AJ237839	DOPEY2
34314_at	X59543	RRM1
37670_at	J04543	ANXA7
38085_at	AI740522	CBX3
828_at	U19487	PTGER2
31873_at	U52112	ARD1A
36847_r_at	AA121509	LSM7
38352_at	AF016371	PPIH
38679_g_at	AA733050	SNRPE
40414_at	X59303	VARS
35318_at	AB007944	FAM20B
37659_at	L42572	IMMT
38760_f_at	U90546	BTN3A2
2025_s_at	M80261	APEX1
41200_at	Z22555	SCARB1
32221_at	AL050361	MRPS18B
1564_at	M63167	AKT1
36876_at	M55150	FAH
36930_at	L05425	GNL2
39713_at	AJ132440	JARID1B
40103_at	X51521	VIL2
35754_at	L40391	TMED10
37730_at	U22055	SND1
38375_at	AF112219	ESD
40979_at	AJ243310	AHSA1
41278_at	AF041474	ACTL6A
1979_s_at	X55504	NOL1
31504_at	M64098	HDLBP
40349_at	AL049442	CBR4
36916_at	X74570	ST3GAL4
38640_at	AI582831	ZNF593
33437_at	AJ005892	FTSJ1
34299_at	AL096880	PATZ1
36647_at	AA526812	IARS2
36673_at	X76057	MPI
33195_at	M94065	DHODH
525_g_at	U13695	PMS1
34647_at	AF015812	DDX5
35467_g_at	W73046	DNAJB12
37794_at	AF035281	LOC791120
38584_at	AF026939	IFIT3
40684_at	U78190	GCHFR
40712_at	D26579	ADAM8
41081_at	AF053305	BUB1
41385_at	AB023204	EPB41L3
41692_at	AB020717	SYNJ1
33748_at	D86976	HMHA1
34719_at	AB020645	GLS

Supplemental Table 2: List of 1462 Transient Genes

34760_at	D14664	CD302
35154_at	W68046	BTBD2
35694_at	AB014587	MAP4K4
35720_at	AB020700	WDR47
36044_at	AF022912	PDE6D
36514_at	U66469	CGRRF1
37228_at	L19559	PLK1
37962_r_at	D63506	STXBP3
39421_at	D43969	RUNX1
39428_at	AF055581	SH2B3
39753_at	X06256	ITGA5
39774_at	X80695	OXA1L
40123_at	D87435	GBF1
40141_at	AB014595	CUL4B
40426_at	X89985	BCL7B
40478_at	AL021396	TXNDC13
40791_at	X63564	POLR2A
40813_at	AI768188	SLC5A3
41183_at	U15782	CSTF3
32776_at	M35416	RALB
41138_at	M16279	CD99
41140_at	U05875	IFNGR2
32169_at	AB020682	FBXO21
35365_at	U40282	ILK
34843_at	AL044599	ZNF516
35254_at	AB007447	TRAFD1
35776_at	AF064243	ITSN1
35811_at	AF037204	RNF13
35833_at	AL080184	INSIG2
36118_at	AJ000882	NCOA1
36139_at	AL050289	TRAF3IP2
36975_at	W26659	TMEM66
37028_at	U83981	PPP1R15A
37326_at	U93305	PLP2
37378_r_at	M13452	LMNA
37652_at	AB002328	CABIN1
37733_at	L35263	MAPK14
37747_at	U05770	ANXA5
38081_at	J03459	LTA4H
38373_g_at	U66042	CXorf40B
38832_r_at	AF053356	GNB2
39091_at	AF070523	ARL6IP5
39809_at	AF019214	HBP1
40220_at	AB021179	HEXIM1
41273_at	AL046940	MXRA7
41808_at	AF052102	DMTF1
1867_at	AF005775	CFLAR
1686_g_at	X82554	SPHAR
1599_at	L25876	CDKN3
1584_at	M34065	CDC25C
1367_f_at	M26880	UBC

Supplemental Table 2: List of 1462 Transient Genes

1257_s_at	L42379	QSOX1
1213_at	U88666	SRPK2
859_at	U03688	CYP1B1
816_g_at	U70987	DOK1
752_s_at	D85429	DNAJB1
493_at	U29171	CSNK1D
178_f_at	U38964	PMS2L8
33800_at	AF036927	ADCY9
34852_g_at	AF011468	AURKA
40631_at	D38305	TOB1
1563_s_at	M58286	TNFRSF1A
621_at	M28211	RAB4A
33700_at	AF039843	SPRY2
37597_s_at	AF055006	EXOC3
32060_at	U15173	BNIP2
37727_i_at	X78669	RCN2
33901_at	U81375	SLC29A1
38372_at	U66042	CXorf40B
1945_at	M25753	CCNB1
38725_s_at	N36295	DPM2
35751_at	U17886	SDHB
39965_at	AI570572	RAC3
41454_at	W27949	HEBP2
36846_s_at	AA121509	LSM7
33439_at	D15050	ZEB1
39852_at	AB011182	SPG20
39210_at	M58597	FUT4
41057_at	AI760162	THEM2
31793_at	AL036554	DEFA1
38282_at	U41767	ADAM15
38683_s_at	AB029008	MASK-BP3
39338_at	AI201310	S100A10
40072_at	Z68747	MRPS31
1827_s_at	M13929	MYC
394_at	X92106	BLMH
39060_at	D38048	PSMB7
39734_at	U10117	SCYE1
32262_at	AL049669	KIAA0859
33415_at	X58965	NME2
34396_at	AB023195	ASXL1
35331_at	U97067	CTNNAL1
39847_at	AF040965	NOL14
1250_at	U47077	PRKDC
33689_s_at	AF012434	DDT
41371_at	Y07595	GTF2H4
32051_at	AJ224875	ALG8
39001_at	AF047470	MDH2
39341_at	AJ001902	TRIP6
41163_at	AL109672	TMED3
34336_at	D32053	KARS
41583_at	AC004770	FEN1

Supplemental Table 2: List of 1462 Transient Genes

32614_at	AB018278	SV2B
1752_at	AD000092	CALR
1476_s_at	U22376	MYB
36446_s_at	L24521	HDGF
31859_at	J05070	MMP9
38978_at	AF013758	PAIP1
39070_at	U03057	FSCN1
40063_at	U22897	CALCOCO2
33817_at	S63912	HNRPA3P1
33433_at	AL049943	FAM98A
36198_at	D13641	TOMM20
36200_at	X69838	EHMT2
32593_at	D42043	RFTN1
631_g_at	L39874	DCTD
31600_s_at	D38435	PMS2L1
31691_g_at	U08997	NA
31699_at	S67334	PIK3CB
33641_g_at	Y14768	AIF1
36447_at	S80990	FCN1
33989_f_at	W28869	TEGT
35385_at	AI766078	COQ7
35465_at	AC003083	SLC25A40
35872_at	U38178	PDE3B
37475_at	AC004144	WDR62
38187_at	D90041	NAT1
41665_at	AB020631	PCF11
41421_at	AB020716	CAMTA2
41662_at	AL050272	NECAP1
32069_at	AB014515	N4BP1
32622_at	L36983	DNM2
33264_at	X89602	ENOSF1
33330_at	U41763	CLTCL1
33770_at	AF009225	CHUK
33776_at	AL050062	RWDD3
35184_at	AB011118	CCDC131
35625_at	X94630	CD97
35693_at	AF070616	HPCAL1
35714_at	U89606	PDXK
36090_at	AL080162	TBL2
37220_at	M63835	FCGR1A
37986_at	M60459	EPOR
38652_at	AF070644	C10orf26
38656_s_at	W27939	TMEM106C
38662_at	AL047596	CIC
38690_at	AL080097	CLDND1
38993_r_at	W27522	TGOLN2
40784_at	Z69030	PPP2R5C
40066_at	AF046024	UBE1C
40069_at	AF051851	SVIL
40130_at	U06863	FSTL1
40137_at	M31724	PTPN1

Supplemental Table 2: List of 1462 Transient Genes

40145_at	AI375913	TOP2A
40473_at	AF024636	STK24
40786_at	U37352	PPP2R5C
41762_at	D64015	TIAL1
32184_at	X61118	LMO2
32857_at	L13858	SOS2
33827_at	AL049783	PFAAP5
32804_at	AF091263	RBM5
33884_s_at	AB014584	UBE4B
33924_at	AB029014	RAB6IP1
35266_at	AL049288	BLCAP
34889_at	AA056747	ATP6V1A
35286_r_at	X76302	RY1
35816_at	U46692	CSTB
36143_at	U13737	CASP3
36146_at	AF057297	OAZ2
36162_at	X64364	BSG
36994_at	M62762	ATP6V0C
37307_at	X04828	GNAI2
38042_at	X03674	G6PD
37383_f_at	L38504	HLA-B
37693_at	L40393	NUMB
37748_at	D86985	KIAA0232
38423_at	L38935	FAM134C
38815_at	Y08999	ARPC1A
38743_f_at	X06409	RAF1
41528_at	W72239	LOC130074
41552_g_at	AW044624	RER1
32592_at	AB002321	KIAA0323
1920_s_at	X77794	CCNG1
1903_at	HG3521-HT3715	NA
1703_g_at	S75174	E2F4
1685_at	X82554	SPHAR
1463_at	M93425	PTPN12
1444_at	AB003177	PSMD9
975_at	Y13115	PLK4
832_at	U39317	UBE2D2
588_at	M31724	PTPN1
418_at	X65550	MKI67
147_at	U82130	TSG101
35218_at	AF022385	PDCD10
40413_at	U18321	DAP3
31526_f_at	X63547	USP6
41716_at	AB020663	DMXL2
1854_at	X13293	MYBL2
969_s_at	X98296	USP9X
39733_at	D14695	HERPUD1
41234_at	AI540318	NA
34546_at	AI250799	DEFA4
38695_at	AF020351	NDUFS4
39704_s_at	L17131	HMGA1

Supplemental Table 2: List of 1462 Transient Genes

37738_g_at	D25547	PCMT1
39040_at	W28360	UBE2J1
40412_at	AA203476	PTTG1
41177_at	AW024285	AYTL2
32255_i_at	U40705	TERF1
32869_at	AF073362	MRE11A
38161_at	Y09022	ALG3
33255_at	M97856	NASP
41759_at	Z47087	SKP1A
38119_at	X12496	GYPC
40588_r_at	AF054186	EEF1E1
2003_s_at	U28946	MSH6
1969_s_at	X77743	CDK7
41868_at	D87002	GGT1
32786_at	X51345	JUNB
1980_s_at	X58965	NME2
1624_at	HG2036-HT2090	NA
1020_s_at	U85611	CIB1
31927_s_at	D86062	C21orf33
41370_at	AF090988	WDR57
31880_at	D83767	UBXD6
33273_f_at	J03011	IGLJ3
33726_at	U89387	POLR2D
35979_at	AF081287	CTDP1
35723_at	D16581	NUDT1
40125_at	L10284	CANX
40129_at	U47077	PRKDC
36624_at	L33842	IMPDH2
37720_at	M22382	HSPD1
38826_at	D50918	39697
39817_s_at	AF040105	C6orf108
374_f_at	Z84718	DDT
36496_at	AF014398	IMPA2
37981_at	U00802	DBN1
37955_at	AB015631	TMEM4
39695_at	M31516	CD55
34368_at	U31814	HDAC2
36968_s_at	AL050353	EXOSC8
37329_at	AF053070	NDUFV1
1242_at	U15655	ERF
947_at	D55716	MCM7
168_at	U50196	ADK
34743_at	D63481	SCRIB
37229_at	U49844	ATR
35801_at	AF026816	ITPA
37031_at	D80005	FAM120A
40584_at	Y08612	NUP88
41838_at	X99270	UCHL5IP
32579_at	D26156	SMARCA4
31788_at	AB014537	ZBED4
36520_at	AB014590	RRP12

Supplemental Table 2: List of 1462 Transient Genes

37648_at	D63487	TTLL12
34353_at	AB014548	PDS5A
37297_at	AL049422	RBM13
38778_at	AB028969	OTUD4
41824_at	AI140114	NME1
31935_s_at	U75968	DDX11
33659_at	X95404	CFL1
32335_r_at	AB009010	UBC
35488_at	U44754	SNAPC1
38523_f_at	D49677	ZRSR2
39931_at	Y12735	DYRK3
39601_at	AF061836	RASSF1
39651_at	AB006532	RECQL4
41387_r_at	AB002344	JMJD3
41467_at	AF070071	MSH5
32120_at	AF063308	SPAG5
41627_at	D50645	SDF2
41651_at	AB028956	KIAA1033
31872_at	X79201	SS18
32059_at	U79282	LOC137886
32621_at	M97388	DR1
32624_at	AL050050	GARNL1
32697_at	AF042729	IMPA1
33741_at	AI741756	ATP6V1H
34210_at	N90866	CD52
32736_at	W68830	RAC2
33256_at	W28626	SLC25A12
33752_at	AB020657	IVNS1ABP
36083_at	U01160	TSPAN31
35688_g_at	Z24459	MTCP1
36492_at	AI347155	WDR66
36530_g_at	AI885381	TRAPPC6A
36542_at	AF030409	SLC9A6
36837_at	U63743	KIF2C
37598_at	D79990	RASSF2
39044_s_at	D73409	DGKD
39335_at	AI074025	EIF5A
39336_at	M74491	ARF3
39714_at	AF042081	SH3BGRL
39411_at	AL080156	TIPARP
39691_at	AB007960	SH3GLB1
39778_at	M55621	MGAT1
40410_at	W26651	DCTN3
40787_at	U90911	WIPF2
40826_at	M80359	MARK3
41127_at	L14595	SLC1A4
41736_g_at	AI808958	DENND3
41766_at	D55649	MAN2A2
33907_at	AF012072	EIF4G3
33346_r_at	M61764	TUBG1
35263_at	N73769	EIF4EBP2

Supplemental Table 2: List of 1462 Transient Genes

34789_at	S69272	SERPINB6
34803_at	AF022789	USP12
34851_at	AF011468	AURKA
35311_at	AF084523	CREG1
35765_at	X91504	ARFRP1
36110_at	M28215	RAB5A
36672_at	L13977	PRCP
37381_g_at	X59268	GTF2B
38431_at	U09759	MAPK9
39552_at	U92436	PTEN
38474_at	L00972	CBS
38739_at	AF017257	ETS2
39896_at	AB011149	DHX16
40278_at	AB029003	GGA2
40898_at	U46751	SQSTM1
40992_s_at	AF055993	SAP30
41333_at	D26069	CENTB2
41561_s_at	AI651368	IFT20
41569_at	AI680675	DNAJC9
32571_at	X68836	MAT2A
32504_at	AW024812	SNX27
32510_at	AF026947	AKR7A2
33120_at	AF045229	RGS10
2093_s_at	J04977	XRCC5
2010_at	U33760	SKP1A
1860_at	U58334	TP53BP2
1846_at	L78132	LGALS8
1817_at	D89667	PFDN5
1315_at	D78361	OAZ1
1238_at	U09759	MAPK9
935_at	L12168	CAP1
1130_at	L11284	MAP2K1
890_at	M74524	UBE2A
809_at	U57094	RAB27A
824_at	U90313	GSTO1
610_at	M15169	ADRB2
503_at	U37690	POLR2L
384_at	X71874	PSMB10
288_s_at	L25931	LBR
181_g_at	S82470	LENG4
35289_at	AJ011679	RABGAP1
38114_at	D38551	RAD21
37980_at	U03644	CIR
38276_at	U91616	NFKBIE
1962_at	M14502	ARG1
1031_at	U09564	SRPK1
39581_at	AA570193	CSTA
34260_at	AB014583	TELO2
34733_at	X85237	SF3A1
38361_g_at	AI688812	RASGRP2
1884_s_at	M15796	PCNA

Supplemental Table 2: List of 1462 Transient Genes

35145_at	X96401	MNT
40420_at	AB015718	STK10
34845_at	AL035398	SAMM50
41681_at	AB005289	ABCB7
32669_at	AB014571	SOCS5
36913_at	U75679	SLBP
40845_at	U10324	ILF3
34393_r_at	AL050268	RAB1A
189_s_at	U09937	PLAUR
32893_s_at	M30474	GGT2
38257_at	AF038406	NDUFS8
39376_at	AB014530	HIPK1
32153_s_at	U49869	UBB
35819_at	X06994	CYC1
37755_at	AB023169	BTBD3
33984_at	M16660	HSP90AB1
37471_at	U94317	RPP40
41696_at	AI620381	C7orf24
38628_at	AF029777	GCN5L2
33934_at	AB018340	SENP6
34829_at	U59151	DKC1
35270_at	W16505	SNRPD2
37046_at	AI246726	PSMA5
36178_at	U23143	SHMT2
36678_at	D21261	TAGLN2
38106_at	AJ012409	TINP1
38768_at	X96752	HADH
39165_at	U47101	ISCU
39814_s_at	AI052724	DHRS7
41812_s_at	AB020713	NUP210
1472_g_at	U22376	MYB
36001_at	Y18643	METTL1
37950_at	X74496	PREP
38353_at	AF042378	TUBGCP3
40764_at	M22632	GOT2
37320_at	D14694	PTDSS1
38792_at	AD001528	SMS
40189_at	M93651	SET
40596_at	U76366	TCOF1
41595_at	AB023164	KIAA0947
41461_at	U13695	PMS1
41415_at	L36720	BYSL
41861_at	AL050019	NOC2L
31836_at	L34600	MTIF2
34259_at	AB014564	KIAA0664
38633_at	U35113	MTA1
39788_at	U81005	PKP4
32151_at	X82260	RANGAP1
32844_at	AF104913	EIF4G1
33409_at	AA158243	FKBP2
35298_at	U54558	EIF3D

Supplemental Table 2: List of 1462 Transient Genes

35312_at	D21063	MCM2
35326_at	AF004876	YIF1A
40255_at	AC004531	DDX28
41831_at	AF077820	LRP5
36727_at	M64936	NA
40476_s_at	U58198	FOXK2
36603_at	D86973	GCN1L1
1188_g_at	X84740	LIG3
31516_f_at	M90354	BTF3L1
31948_at	X79563	RPS21
33602_at	AJ000479	EDG6
36444_s_at	AF088219	CCL23
36781_at	X01683	SERPINA1
38492_at	D55639	KYNU
40669_at	U57094	RAB27A
41083_at	AC006276	ZNF428
34721_at	U42031	FKBP5
37617_at	U90912	KIAA1128
38340_at	AB014555	HIP1R
38653_at	D11428	PMP22
32750_r_at	X53416	FLNA
37001_at	M23254	CAPN2
38078_at	AF042166	FLNB
32541_at	S46622	PPP3CC
2011_s_at	U34584	BIK
40012_at	D86407	LRP8
33162_at	X02160	INSR
34378_at	X97324	ADFP
40391_at	AB007448	SLC22A4
36980_at	U03105	PNRC1
38767_at	AF041037	SPRY1
37904_s_at	X66436	GNL1
1274_s_at	L22005	CDC34
38997_at	X96924	SLC25A1
39772_at	AF007157	PRNPIP
1527_s_at	U50527	CG018
37162_at	S72869	CCDC6
34261_at	D84307	PCYT2
34470_at	D43945	TFEC
40018_at	AB007870	NUPL1
40385_at	U64197	CCL20
41619_at	AL022398	IRF6
35668_at	AJ001014	RAMP1
35718_at	L22342	SP110
40836_s_at	W26677	EML3
36657_at	AA883870	APOC2
38458_at	L39945	CYB5A
40964_at	Z46369	HK2
1790_s_at	HG3914-HT4184	NA
1642_at	U35113	MTA1
447_g_at	U89896	CSNK1G2

Supplemental Table 2: List of 1462 Transient Genes

41232_at	AL050022	TCTN3
32118_at	AF076838	RAD17
38513_at	D31765	POP1
33744_at	AL080150	GEMIN4
34300_at	AI352450	PATZ1
37713_at	L07548	ACY1
41790_at	AL031230	ALDH5A1
38241_at	U90548	BTN3A3
38917_at	X73617	TRA@
41442_at	AB010419	CBFA2T3
33724_at	U64805	BRCA1
38694_at	AB018281	FAM115A
39786_at	AF070525	GRPEL1
41503_at	AB020661	ZHX2
37347_at	AA926959	CKS1B
38251_at	X16434	MYL6B
33849_at	U02020	PBEF1
1162_g_at	HG3227-HT3404	NA
31814_i_at	AB009462	LRP3
32679_at	D13634	MTFR1
33232_at	AI017574	CRIP1
33274_f_at	X79783	IGL@
34734_at	X71661	LMAN1
38966_at	AF038958	GPSN2
38436_at	D87440	RTF1
38757_at	U41745	PDAP1
40591_at	S78234	CDC27
1480_at	L12723	HSPA4
1410_at	J03258	VDR
37391_at	X12451	CTSL1
39556_at	M96803	SPTBN1
1065_at	U02687	FLT3
37121_at	S69115	NKG7
2061_at	L12002	ITGA4
38911_at	U41815	NUP98
32402_s_at	Y10931	SYMPK
37416_at	Z35227	RHOH
39258_at	AI627877	RNF126
32974_at	U07563	EXOSC2
40032_at	D50923	KIAA0133
38454_g_at	X15606	ICAM2
34482_at	AF040964	C4orf15
38201_at	U21551	BCAT1
31815_r_at	AB009462	LRP3
34263_s_at	Y15908	DIAPH2
35672_at	AL080144	AHCTF1
39835_at	U93181	SBF1
39883_at	AF091078	DIMT1L
41001_at	AB023202	RPH3A
41509_at	L11066	HSPA9
33206_at	C18655	ABBA-1

Supplemental Table 2: List of 1462 Transient Genes

1376_at	M36067	LIG1
31842_at	AF038195	BCS1L
39729_at	L19185	PRDX2
37838_at	M31315	F12
37749_at	D78611	MEST
31605_at	U72518	LOC171220
39653_at	AL050120	POT1
36566_at	AJ222967	CTNS
41149_at	AC004381	LOC81691
41775_at	AF064084	ICMT
36205_at	L04490	NDUFA9
37360_at	U66711	LY6E
1717_s_at	U45878	BIRC3
36689_at	AL040446	OSBPL1A
38218_at	M97347	GCNT1
41352_at	AF007133	ST6GAL1
1822_at	HG4677-HT5102	NA
35071_s_at	AF042377	GMDS
34190_at	AF006740	COCH
35657_at	U08998	TARBP2
36462_at	U50383	SMYD5
38007_at	L11353	NF2
38726_at	W80399	DPM2
40973_at	AI146846	PARD3
40280_at	U72508	LRRC23
1590_s_at	J00277	HRAS
39387_at	U34044	SEPHS1
40183_at	AI660225	YIPF2
1462_s_at	M80397	POLD1
34583_at	U02687	FLT3
32213_at	AA203527	POP7
37649_at	M95623	HMBS
40936_at	AI651806	CRIM1
36891_at	AL022237	MCAT
37993_at	X63422	ATP5D
35792_at	U67963	MGLL
2057_g_at	M34641	FGFR1
33202_f_at	U43747	FXN
37225_at	D79994	ANKRD15
2020_at	M73554	CCND1
1071_at	M77810	GATA2
37832_at	AL080062	MMACHC
34805_at	AA195301	CCDC86
160026_at	X85545	PRKX
31392_r_at	U88965	C3orf51
31625_at	AB007921	C1orf34
31438_s_at	Z22971	CD163
31524_f_at	Z80782	HIST1H2BI
31783_at	U52112	RENBP
32389_at	W25892	RNU2
32475_at	AF025529	LILRA1

Supplemental Table 2: List of 1462 Transient Genes

33034_at	Y17108	RHBDL1
33042_r_at	AF055018	FAM55C
33647_s_at	AA224768	GM2A
34083_at	AA311181	15E1.2
35016_at	M13560	CD74
35114_at	AF084645	NR1I2
36377_at	U43672	IL18R1
36400_at	AA831707	PRIM1
36412_s_at	U53831	IRF7
32355_at	AL050270	TANC2
32977_at	U49187	C6orf32
33462_at	D13626	P2RY14
33487_at	D31628	HPD
33535_at	U45448	P2RX1
33538_at	H96671	MYEF2
33954_at	AL049988	ITPR2
33956_at	AB018549	LY96
33983_at	Z33642	IGSF2
34977_at	AJ007395	SIGLEC7
34478_at	X79780	RAB11B
34498_at	D89974	VNN2
34509_at	AF016833	MGAM
34946_at	AJ223183	IGSF6
35000_at	U03398	TNFSF9
35892_at	Y00816	CR1
36223_at	AI809970	SFPQ
36243_at	AL050262	TLR1
36255_at	L77730	ADORA3
36324_at	X68487	ADORA2B
36709_at	Y00093	ITGAX
36724_s_at	U95626	CCR5
36759_at	U29589	CHRM3
37068_at	U24577	PLA2G7
37112_at	AB002384	C6orf32
37114_at	L32832	ZFHX3
38167_at	AB020704	PPFIA4
38576_at	AJ223353	HIST1H2BD
37148_at	AF025533	LILRB3
37489_s_at	U05596	SLC4A3
37490_at	L27213	SLC4A3
37769_at	AF011466	EDG4
37835_at	M28827	CD1C
38131_at	AF010316	PTGES
38207_at	AW006742	TncRNA
38490_r_at	U00944	CXorf45
38567_at	L38820	CD1D
38879_at	D83664	S100A12
39301_at	X85030	CAPN3
39313_at	AB002342	WNK1
39925_at	M95610	COL9A2
40648_at	U08023	MERTK

Supplemental Table 2: List of 1462 Transient Genes

39603_at	AB007926	DISC1
40323_at	D84276	CD38
40656_at	D85730	HSPA1L
40700_at	U36500	SP140
40729_s_at	Y14768	LTB
41059_at	AF020314	CD300A
41714_at	AB007924	LPPR4
41863_at	AF070623	RALYL
32026_s_at	AB002311	RAPGEF2
32083_at	AF027826	GPR137B
33811_at	AI761567	CCPG1
32716_at	AF064768	DGKA
33224_at	AB007965	CYHR1
33236_at	AF060228	RARRES3
33777_at	D34625	TBXAS1
34224_at	AC004770	FADS3
34256_at	AB018356	ST3GAL5
34714_at	AL050267	SAMHD1
35160_at	AF064491	LDB1
35650_at	AB002354	PLEKHM1
36022_at	AB007942	DNAJC6
36082_at	S71326	CEACAM1
35626_at	U30894	SGSH
35674_at	AB023211	PADI2
35686_s_at	Z24459	MTCP1
35692_at	AL080235	TMEM158
36004_at	AF074382	IKBKG
36059_at	AB011540	LRP4
36089_at	AB023183	INPP5F
36464_at	X94323	CRISP3
36825_at	X82200	TRIM22
37603_at	X52015	IL1RN
37970_at	AB028989	MAPK8IP3
37976_at	AL034397	VSIG4
37929_at	AB017563	CADM1
38291_at	J00123	PENK
38325_at	AL050356	MINPP1
38336_at	AB023230	FRMD4B
38721_at	W72733	CALCOCO1
38968_at	AB005047	SH3BP5
39054_at	X08020	GSTM1
39706_at	AB014536	CPNE3
40782_at	AF061741	DHRS3
39361_f_at	AF043906	TSPAN6
39432_at	AF038662	B4GALT4
39771_at	AB018283	RHOBTB1
40046_r_at	AF009426	C18orf1
40049_at	X76104	DAPK1
40155_at	D31883	ABLIM1
40519_at	Y00638	PTPRC
40520_g_at	Y00638	PTPRC

Supplemental Table 2: List of 1462 Transient Genes

40815_g_at	L40586	IDS
40825_at	AB025186	MAPRE3
41191_at	AB023209	PALLD
32156_at	AF044968	PVRL2
40863_r_at	X15334	CKB
32176_at	AB011110	RASA4
32200_at	M24902	ACPP
32217_at	AF052105	FAM130A1
32747_at	X05409	ALDH2
32765_f_at	W28330	PGCP
33366_at	AL022238	SGSM3
34339_at	AB009282	CYB5B
35350_at	AB011170	GALNAC4S-6ST
34784_at	Z83844	TRIOBP
34844_at	D86975	ZNF516
35287_at	AF046888	TNFSF13
35755_at	U51336	ITPK1
35829_at	AL080181	CADM1
36618_g_at	X77956	ID1
36977_at	U39412	NAPA
37303_at	AF057160	PARP4
37706_at	U28811	GLG1
37687_i_at	M31932	FCGR2A
37760_at	AB015019	BAIAP2
37767_at	Z49155	HD
38021_at	U53204	PLEC1
38107_at	U40998	UNC119
38404_at	M55153	TGM2
38735_at	AB011085	KIAA0513
39545_at	U22398	CDKN1C
39833_at	R54564	MINK1
39837_s_at	AC004877	ZNF467
38740_at	X79067	ZFP36L1
38799_at	AF068706	AP1G2
39893_at	AB010414	GNG7
40268_at	X16706	FOSL2
40175_at	AI141670	FAM131A
40539_at	U42391	MYO9B
40626_at	AI693193	MTX1
40913_at	W28589	ATP2B4
41257_at	D16217	CAST
41323_at	U90917	FOXM1
41493_at	AI094610	ATP11A
41518_at	AI925356	YKT6
32533_s_at	AF054825	VAMP5
2017_s_at	M64349	CCND1
2019_s_at	M68892	ITGB7
1779_s_at	M16750	PIM1
1786_at	U08023	MERTK
1729_at	L41690	TRADD
1671_s_at	L35253	MAPK14

Supplemental Table 2: List of 1462 Transient Genes

1649_at	U61836	SMOX
1650_g_at	U61836	SMOX
1575_at	M14758	ABCB1
1553_r_at	U22028	CYP2A13
1491_at	M31166	PTX3
1375_s_at	M32304	TIMP2
1232_s_at	M74587	IGFBP1
1158_s_at	J04046	CALM3
1120_at	J05459	GSTM3
1061_at	U00672	IL10RA
1062_g_at	U00672	IL10RA
988_at	X16354	CEACAM1
973_at	Y10032	SGK
944_s_at	D49354	PPFIA1
874_at	M26683	CCL2
858_at	S90469	POR
294_s_at	HG4120-HT4392	NA
753_at	D86425	NID2
669_s_at	L05072	IRF1
599_at	M60721	HLX
556_s_at	M96233	GSTM4
544_at	S76638	NFKB2
496_s_at	U32324	IL11RA
491_at	U46116	PTPRG
484_at	U59302	NCOA1
449_at	U66469	CGRRF1
366_s_at	Z29066	NEK2
370_at	Z35102	STK38
371_at	Z56281	IRF3
203_at	M68891	GATA2
268_at	L34657	PECAM1
245_at	M25280	SELL
177_at	U38545	PLD1
160041_at	X79568	PTPN18
160022_at	X03663	CSF1R
