# Supplementary material for: Decomposition of Gene Expression State Space Trajectories
Source: PLoS Comput Biol. 2009 Dec 24;5(12):e1000626. doi: 10.1371/journal.pcbi.1000626 (PMC2791157; doi:10.1371/journal.pcbi.1000626)
Supplement: Text S1 — The Likelihood Ration Test. Text S1 contains information on how the likelihood ratio test and the analysis of deviance test are computed from the data. This section includes an example using expression data for a gene to show how our method uses these tests to places genes in the core and transient groups. (0.04 MB PDF) [file pcbi.1000626.s010.pdf]

Text S1: Computing the Likelihood Ratio Statistic and the Analysis of Deviance from the Data

Inherent in our linear model is the assumption that the gene expression values under ATRA and DMSO induction follow a Normal distribution. The probability density function *f* of an expression value at time point *j* is given by:

$$f(y_{ATRA,j}) = \frac{1}{\sqrt{2\pi\sigma^2}} \exp\left\{-\frac{(y_{ATRA,j} - \mu_{ATRA,j})^2}{2\sigma^2}\right\}$$

$$f(y_{DMSO,j}) = \frac{1}{\sqrt{2\pi\sigma^2}} \exp\left\{-\frac{(y_{DMSO,j} - \mu_{DMSO,j})^2}{2\sigma^2}\right\}$$

for $j = 1, \ldots, 12$.

The full and reduced models are equivalent to imposing different equations for the expression means at each time point: $\mu_{ATRA} = (\mu_{ATRA,1},\ldots,\mu_{ATRA,12})$ and $\mu_{DMSO} = (\mu_{DMSO,1},\ldots,\mu_{DMSO,12})$.

Under the full model $\theta_{full} = (\mu_{ATRA}, \mu_{DMSO})$:

where $\mu_{ATRA,j} = \alpha_0 + \alpha_1 t_j + \alpha_2 t_j^2 + \alpha_3 t_j^3$ and $\mu_{DMSO,j} = \beta_0 + \beta_1 t_j + \beta_2 t_j^2 + \beta_3 t_j^3$.

Under the reduced model $\theta_{reduced} = (\mu_{ATRA}, \mu_{DMSO})$:

where $\mu_{ATRA,j} = \mu_{DMSO,j} = \lambda_0 + \lambda_1 t_j + \lambda_2 t_j^2 + \lambda_3 t_j^3$.

For a single gene *g*, the log-likelihood function for its expression profile is given by:

$$l(Y_g \mid \mu_{ATRA}, \mu_{ATRA}, \sigma^2) = \sum_{j=1}^{N} \log f(y_{ATRA,j}) + \sum_{j=1}^{N} \log f(y_{DMSO,j})$$
$$= -N \log(2\pi\sigma^2) - \left[\sum_{j=1}^{N} \frac{(y_{ATRA,j} - \mu_{ATRA,j})^2 + (y_{DMSO,j} - \mu_{DMSO,j})^2}{2\sigma^2}\right]$$

where $N = 12$.

When the likelihood ratio statistic is used as a goodness of fit test between models, the software R actually carries out an analysis of deviance, which is an extension of the likelihood ratio test. The analysis of deviance is valid as a goodness of fit test for all classes of linear models, including generalized linear models and mixed effects models.

The deviance is defined as a linear function of the log-likelihood function:

$$\frac{D(Y_g;\theta_{full})}{\phi} = 2l(Y_g \mid \theta) - 2l(Y_g \mid \theta_{full})$$

where $\phi = \sigma^2$ for the Normal distribution.

The likelihood ratio statistic is simply the difference in scaled deviances between the reduced and full models:

$$\frac{D(Y_g;\theta_{red}) - D(Y_g;\theta_{full})}{\phi} = -2\left[l(Y_g \mid \theta_{red}) - l(Y_g \mid \theta_{full})\right] \sim \chi^2_4$$

For the Normal distribution, the scaled deviances correspond to the residual sum of squares (RSS) that are obtained by fitting the reduced and full models to the expression profile data.

$$RSS_{red} - RSS_{full} = -2\left[l(Y_g \mid \theta_{red}) - l(Y_g \mid \theta_{full})\right] \sim \chi^2_4$$

Because the full model specifies more parameters, automatically $RSS_{full} \leq RSS_{red}$ and the model fit or likelihood will be higher under the full model, compared to the reduced model. What we are interested in testing then is whether the improvement in fit obtained with the full model over the reduced model is statistically significant.

We compare the ratio between the likelihood ratio statistic (and its degrees of freedom) and the RSS_{full} (and its degrees of freedom):

$$F = \frac{\left(RSS_{red} - RSS_{full}\right)/4}{RSS_{full}/16} \sim F_{4,16}\,.$$

The rationale is that when the full model results in a very large improvement in model fit over the reduced model, $RSS_{red} - RSS_{full}$ will be very large and subsequently the ratio F will also be very large. The corresponding P-value will be very small (and after adjusting for multiple testing is also likely to be still small enough to satisfy the significance threshold), hence the gene will be placed in the transient group. Alternatively, if the improvement associated with the full model is negligible, then $RSS_{red} - RSS_{full}$ will be close to zero and the ratio F will be small. The corresponding adjusted P-value will be close to 1, and the gene will be placed in the core group.

Here we provide some R code to illustrate how we fit the reduced and full models to expression data for a single gene and also how we use the analysis of deviance test to evaluate which model provides the best fit. As an example, we use the expression profile data for probeset "36209_at" which maps to the BRD2 gene (Entrez GeneID: 6046).

```
> Y.dmso <- c(-0.2,-0.6,-0.7,-0.9,-0.4,-0.8,-1.8,0.4,-1.8,-0.6,-0.8,-1.0)
> Y.atra <- c(-1.4,-0.9,-1.0,-1.6,-3.0,-0.3,-0.4,0.2,-0.1,-4.2,-0.3,-4.9)
> Y.core <- c(Y.dmso, Y.atra)
```

The `timeX` object specifies the time points in hours:

```
> timeX <- c(2, 4, 8, 12, 18, 24, 48, 72, 96, 120, 144, 168)
```

We create the design matrices `X.full` and `X.mini` that specify the full and reduced models, respectively:

```
> Xd <- cbind(rep(1,12), timeX, timeX^2, timeX^3, matrix(0, nrow=12, ncol=4))
> Xr <- cbind(matrix(0, nrow=12, ncol=4), rep(1,12), timeX, timeX^2, timeX^3)
> X.full <- rbind(Xd, Xr)
> X.mini <- cbind(rep(1,24), rep(timeX,2), rep(timeX^2,2), rep(timeX^3,2))
> colnames(X.full) <- c(paste("a", 0:3, sep=""), paste("b", 0:3, sep=""))
> colnames(X.mini) <- paste("d", 0:3, sep="")
```

Fitting the linear models for the full and reduced models:

```
> fullMod.core <- lm(Y.core ~ a0 + a1 + a2 + a3 + b0 + b1 + b2 + b3 - 1,
+ dat=data.frame(X.full))
> miniMod.core <- lm(Y.core ~ d0 + d1 + d2 + d3 - 1, dat=data.frame(X.mini))
```

To test the goodness of fit between the reduced and the full models:

```
> anova(miniMod.core, fullMod.core)
```

and the following output is obtained:

```
Model 1: Y.core ~ d0 + d1 + d2 + d3 - 1
Model 2: Y.core ~ a0 + a1 + a2 + a3 + b0 + b1 + b2 + b3 - 1
  Res.Df     RSS Df Sum of Sq      F Pr(>F)
1     20 31.5335
2     16 22.5473  4    8.9862 1.5942 0.2241
```

Here $RSS_{red} = 31.5335$ and $RSS_{full} = 22.5473$, hence $RSS_{red} - RSS_{full} = 8.9862$ and the F statistic is given by:

$$F = \frac{\left(RSS_{red} - RSS_{full}\right)/4}{RSS_{full}/16} = \frac{8.9862/4}{22.5473/16} = 1.5942$$

Since the probability of observing this F statistic or something more extreme under the $F_{4,16}$ distribution is large ($P = 0.2241$) we conclude that the reduced model is an adequate fit over the full model. This gene would be classified as a core gene (our significance threshold was set at 0.1after adjustment for multiple correction testing). Note that for the 3841 genes, after adjusting all 3841 P-values using the Benjamini-Hochberg correction method, the adjusted P-value for this gene was 0.3733.

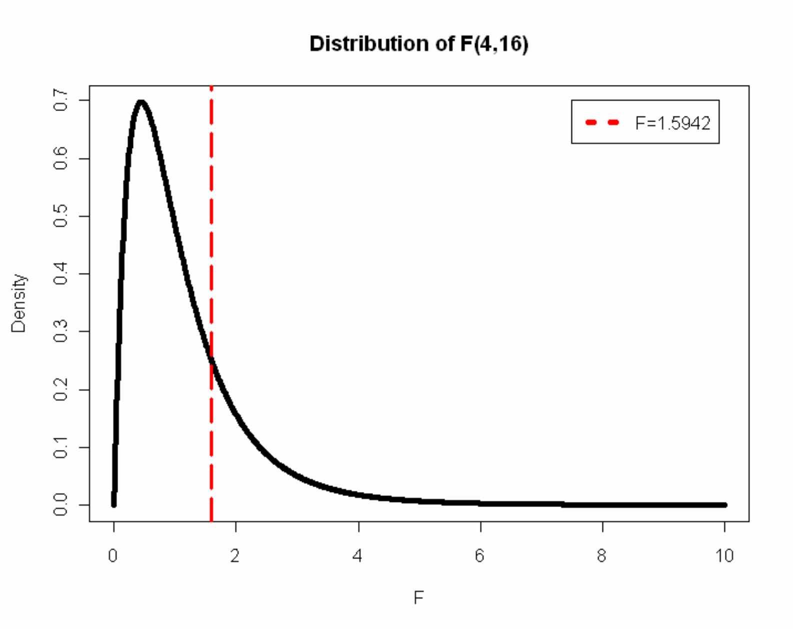
Distribution of F(4,16)
F=1.5942
Density
F
0.0
0.1
0.2
0.3
0.4
0.5
0.6
0.7
0
2
4
6
8
10
