# Supplementary material for: Decomposition of Gene Expression State Space Trajectories
Source: PLoS Comput Biol. 2009 Dec 24;5(12):e1000626. doi: 10.1371/journal.pcbi.1000626 (PMC2791157; doi:10.1371/journal.pcbi.1000626)
Supplement: Text S2 — Using Fisher's exact test. Text S2 outlines how we use the Fisher's exact test to compute the significance of seeing an enrichment of transient or core genes in sets of genes extracted from the literature. (0.02 MB PDF) [file pcbi.1000626.s011.pdf]

Text S2: Computing the Fisher's Exact Test to Assess Overenrichment

For the 10 stimulus-induced genes by ATRA or DMSO (PTEN, Akt1, p27, RXR-α, p21, CITED2, RARRES3, MBN, CD38, SMYD5), 9 out of 10 were classified as transient genes.

	**Stimulus-Induced Genes**	**Non-Stimulus Induced Genes**	
**Transient Genes**	$X = 9$	1461	$k = 1462$
**Core Genes**	1	1419	$N - k = 1428$
	$M = 10$		$N = 2890$

The quantity X can be modeled as a hypergeometric random variable:

$$P(X = x) = \frac{\binom{M}{x}\binom{N-M}{k-x}}{\binom{N}{k}}$$

The statistical significance of seeing 9 out of 10 stimulus-induced genes represented in the transient group of genes corresponds to calculating the sum of probabilities greater than equal to what we observed.

$$P(X \geq 9) = \sum_{x=9}^{10} \frac{\binom{M}{x}\binom{N-M}{k-x}}{\binom{N}{k}} = 0.0117.$$

For the sixteen genes identified by [15, 16], thirteen were classified as core genes.

	**Myeloid-Specific Differentiation Genes**	**Non-Myeloid-Specific Differentiation Genes**	
**Core Genes**	$X = 13$	1415	$k = 1428$
**Transient Genes**	3	1459	$N - k = 1462$
	$M = 16$		$N = 2890$

The statistical significance of seeing 13 out of 16 myeloid-specific differentiation genes represented in the core group of genes corresponds to calculating the sum of probabilities greater than equal to what we observed.

$$P(X \geq 13) = \sum_{x=13}^{16} \frac{\binom{M}{x}\binom{N-M}{k-x}}{\binom{N}{k}} = 0.00924.$$

Similarly, for the apoptosis-related genes:

	**Apoptosis-Related Genes**	**Non-Apoptosis-Related Genes**	
**Core Genes**	$X = 10$	1418	$k = 1428$
**Transient Genes**	3	1459	$N - k = 1462$
	$M = 13$		$N = 2890$

$$P(X \geq 10) = \sum_{x=13}^{13} \frac{\binom{M}{x}\binom{N-M}{k-x}}{\binom{N}{k}} = 0.0418.$$
